# Efficacy and safety of ofatumumab in participants with relapsing multiple sclerosis and breakthrough disease on oral fingolimod or fumarates: results from the ARTIOS study

**DOI:** 10.1007/s00415-026-13960-5

**Published:** 2026-06-29

**Authors:** Riley Bove, Dawn Langdon, Maciej Maciejowski, Elżbieta Jasińska, Michal Dufek, Anil Abeyewickreme, Anamaria Rauh, Haoyi Fu, Imran Ali Khan, Matthias Böhringer, Sara Madueno Eichau, Tobias Derfuss

**Affiliations:** 1 University of California San Francisco, San Francisco, CA USA; 2 Royal Holloway, University of London, London, UK; 3 MA-LEK, MS Center, Katowice, Poland; 4 Collegium Medicum, Jan Kochanowski University, Kielce, Poland; 5 First Department of Neurology, St. Anne’s University Hospital, Masaryk University, Brno, Czechia; 6 Novartis Pharmaceuticals UK, London, UK; 7 Novartis Pharma AG, Basel, Switzerland; 8 Novartis Pharmaceuticals Corporation, East Hanover, NJ USA; 9 Novartis Healthcare Private Limited, Hyderabad, India; 10 Neurozentrum Bielefeld, Bielefeld, Germany; 11 Department of Neurology, Hospital Universitario Virgen Macarena, Seville, Spain; 12 Neurology Clinic and Policlinic and Research Center for Clinical Neuroimmunology and Neuroscience, Departments of Medicine and Biomedicine, University Hospital and University of Basel, Basel, Switzerland

**Keywords:** Relapsing Multiple Sclerosis (RMS), Ofatumumab, Treatment Switch, Disease-Modifying Therapies (DMTs), Fingolimod, Fumarates

## Abstract

**Background:**

Initiating high-efficacy disease-modifying therapies (DMTs) early in relapsing multiple sclerosis (RMS) can reduce inflammation and limit disability progression; however, moderate-efficacy oral DMTs remain common first-line treatments. Ofatumumab demonstrated superior efficacy and tolerable safety in the phase 3 ASCLEPIOS trials, although few participants (≤ 5%) transitioned from oral DMTs. ARTIOS evaluated the efficacy and safety of ofatumumab in adults with RMS switching from fingolimod or fumarates following breakthrough disease.

**Methods:**

ARTIOS was a phase 3b, open-label, single-arm, multicenter, noncomparative study. Primary endpoint was annualized relapse rate (ARR); secondary endpoint was safety.

**Results:**

562 adults on fingolimod (n = 181) or fumarates (n = 381) with breakthrough disease, defined as ≥ 1 relapse in prior year or ≥ 2 relapses in prior 2 years and/or magnetic resonance imaging (MRI) evidence of disease activity in prior year, were enrolled. The primary endpoint was met, with a low ARR overall (0.06; 95% CI: 0.05–0.08; p < 0.0001) and by prior DMT (fingolimod: 0.09; 95% CI: 0.06–0.1; fumarates: 0.06; 95% CI: 0.04–0.08), despite greater baseline disease severity with fingolimod. Ofatumumab resulted in near-complete suppression of MRI lesions, and 90.9% of participants achieved no evidence of disease activity, regardless of prior DMT. Six-month confirmed disability worsening occurred in few participants (7.3%). Safety outcomes were consistent with those of prior studies; most treatment-emergent adverse events (TEAEs) were mild to moderate, serious TEAEs were uncommon (5.9%), and rates of treatment discontinuations and interruptions were low.

**Conclusions:**

ARTIOS complements the ASCLEPIOS studies and supports ofatumumab following switch from oral DMTs.

**Trial registration:**

ClinicalTrials.gov, NCT04353492; April 20, 2020.

**Supplementary Information:**

The online version contains supplementary material available at 10.1007/s00415-026-13960-5.

## Introduction

Multiple sclerosis (MS) specialists have shifted toward initiating high-efficacy therapies (HETs) early in disease to optimize patient outcomes, including reduced inflammatory activity, neuronal injury, and disability [1–5]. Across prospective and retrospective studies, interventional trials and observational real-world studies in MS, early initiation of HETs, such as anti-CD20 monoclonal antibodies, improves long-term outcomes by reducing inflammatory activity and slowing disability progression [6–11]. Despite these findings, moderate-efficacy oral therapies, such as fingolimod or fumarates, are still often used as first-line treatments, due to being perceived as safer [1, 4]. If disease is not well controlled on such treatments, early switch to HETs can help avoid further central nervous system damage and may be preferable to cycling between lower-efficacy therapies, which remains common but can increase risk of disability worsening and progression [1, 5].

Ofatumumab, a fully human anti-CD20 monoclonal antibody with a 20 mg subcutaneous monthly dosage regimen, is the only self-administered anti-CD20 therapy approved for the treatment of adults with relapsing MS (RMS). Approval was based on results of the ASCLEPIOS phase 3 trials, which established the favorable benefit-risk profile of ofatumumab versus teriflunomide in RMS [12–15]. However, in these pivotal studies, ≤ 5% of participants had been previously treated with oral therapies. Therefore, it was important to better understand outcomes in participants switching to ofatumumab after oral therapy [16, 17]. This article reports findings from ARTIOS (NCT04353492), a phase 3b study to assess the efficacy and safety of ofatumumab in participants with RMS transitioning from fingolimod or fumarates due to breakthrough disease activity.

## Methods

### Study design

ARTIOS was a phase 3b, open-label, single-arm, multicenter, non-comparative, 96-week study of ofatumumab 20 mg administered subcutaneously every 4 weeks in participants with RMS who experienced breakthrough disease (defined in “Participants” section) while being treated with fingolimod or fumarates. The study was designed to allow alignment with locally approved ofatumumab labels and standard medical practice, providing investigators flexibility in assessments such as prior disease-modifying therapy (DMT) washout duration, symptoms assessment, and observation time at the site.

**Fig. 1:**
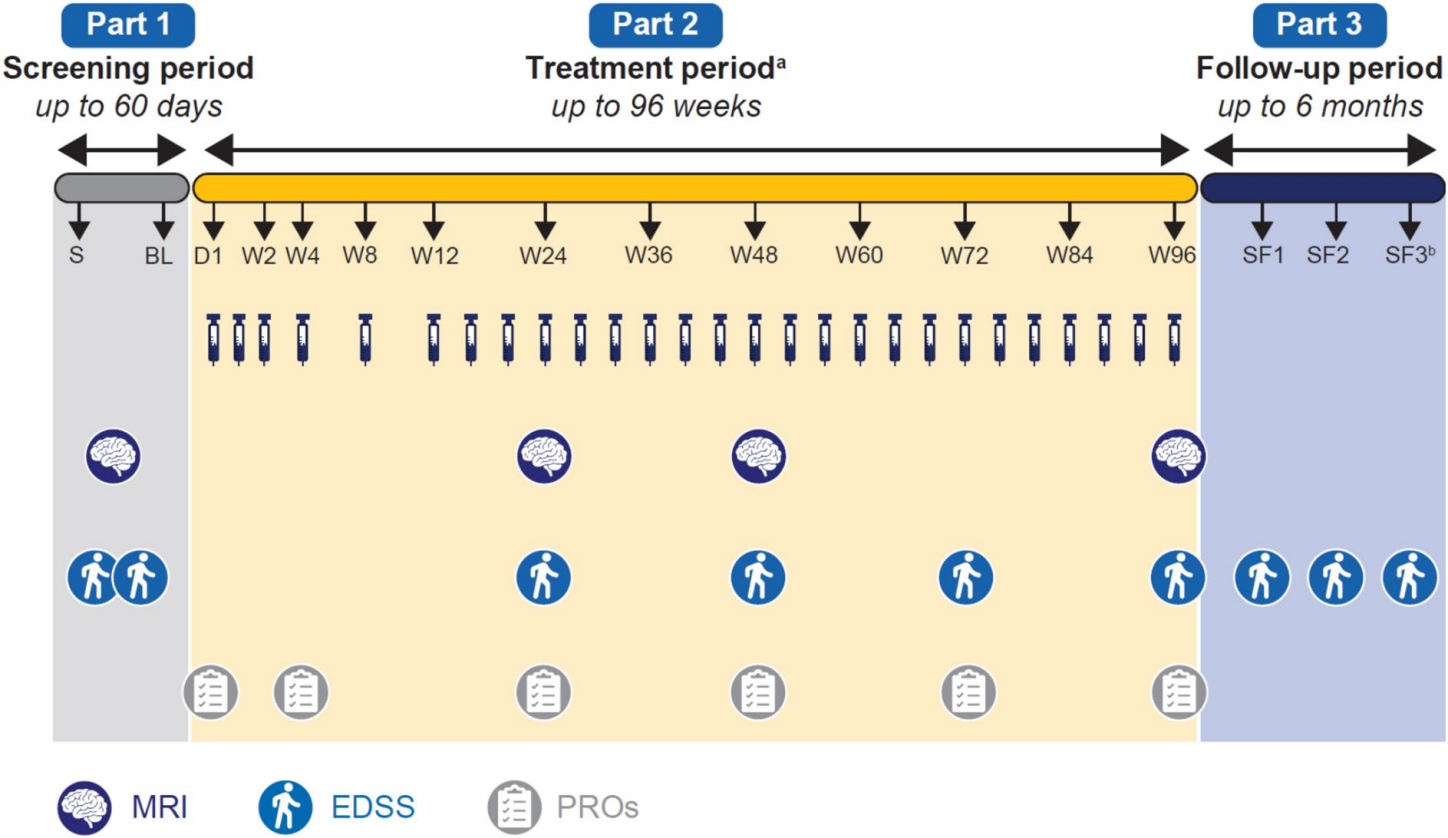
ARTIOS study design**.** ^a^ Ofatumumab 20 mg was administered subcutaneously every 4 weeks following an initial loading regimen of three 20 mg subcutaneous doses over the first 14 days (administered on Days 1, 7, and 14). ^b^ Additional follow-up visits occurred every 3 months (± 14 days) after the second safety follow-up visit (SF2). Participants who prematurely discontinued during the treatment period, as well as those who completed the study but did not continue ofatumumab treatment, entered the safety follow-up period**.** *BL* baseline; *D* day; *EDSS* Expanded Disability Status Scale; *MRI* magnetic resonance imaging; *PRO* patient-reported outcome; *S* screening; *SF* safety follow-up visit;* W* week

The study consisted of three periods (Fig. 1). The screening period (Part 1) lasted up to 60 days and included the transition phase, during which the exact timing between discontinuation of prior DMT and initiation of ofatumumab was determined by investigator’s clinical judgement. During this period, participants were not permitted to receive any other DMTs. The treatment period (Part 2) consisted of induction and maintenance phases over 96 weeks. During the induction phase (Weeks 1–4), participants received subcutaneous ofatumumab 20 mg via autoinjector on Days 1, 7, and 14. Induction was followed by a maintenance phase in which participants received subcutaneous ofatumumab 20 mg every 4 weeks, starting at Week 4. The safety follow-up period (Part 3) lasted ≤ 6 months and applied to participants who completed the treatment period without continuing ofatumumab post-trial and those who prematurely discontinued treatment during the study.

The ARTIOS protocol was reviewed and approved by an Institutional Review Board/Independent Ethics Committee at each participating site (Online Resource Table 1). ARTIOS was conducted in accordance with the Declaration of Helsinki and Council for International Organizations of Medical Sciences international ethical guidelines, applicable ICH Good Clinical Practice guidelines, and all applicable laws and regulations.

### Participants

All participants provided written informed consent. Enrolled participants included adults (aged 18–60 years) with RMS, including active secondary progressive MS [18], diagnosed according to the 2017 revised McDonald criteria [19]; an Expanded Disability Status Scale (EDSS) score of 0 to 4 at screening; prior treatment with a maximum of 3 DMTs; and transitioning from either fingolimod or fumarates administered for ≥ 6 months as their last prior DMT. Participants had to have breakthrough disease activity while receiving adequate treatment with fingolimod or fumarates for ≥ 6 months prior to transitioning. Breakthrough disease was defined as ≥ 1 documented relapse in the previous year or ≥ 2 relapses in previous 2 years; ≥ 1 gadolinium-enhancing (Gd +) magnetic resonance imaging (MRI) lesion within the last year; or new or enlarging T2 lesions (neT2) within the last year. Full inclusion and exclusion criteria are listed in the **Supplementary Information**.

### Endpoints and assessments

The primary endpoint was the annualized relapse rate (ARR), defined as the number of confirmed MS relapses measured over 96 weeks. The secondary endpoint was safety, including assessment of treatment-emergent adverse events (TEAEs), injection-related reactions (IRRs), and clinical laboratory evaluations, including mean immunoglobulin (Ig) G and IgM levels, and the proportion of participants discontinuing treatment due to insufficient effectiveness or for tolerability or safety reasons.

Exploratory efficacy assessments included time to 6-month confirmed disability worsening (6mCDW), defined as an increase from baseline in EDSS score sustained for 6 months; number of T1 Gd + lesions per scan, number of neT2 lesions per year, and proportion of participants with no evidence of disease activity (NEDA-3) during the 96-week period, a composite endpoint defined as no confirmed relapses, no neT2 lesions compared with baseline, no T1 Gd + lesions, and no 6mCDW. Additional exploratory endpoints included timed 25-foot walk (T25FW), nine-hole peg test (9HPT), symbol digit modalities test (SDMT), low-contrast visual acuity (LCVA), time to 6-month confirmed cognitive decline (6mCCD), serum biomarker neurofilament light chain (NfL) values, T2 lesion volume, and proportion of participants without MRI activity. Patient-reported outcome (PRO) assessments of the impact of MS disease included change from baseline in the Multiple Sclerosis Impact Scale 29 (MSIS-29), change from baseline on the Fatigue Scale for Motor and Cognitive Functions (FSMC), and change from baseline on the Treatment Satisfaction Questionnaire for Medication (TSQM 1.4).

### Statistical analysis

The full analysis set (FAS) included all study participants who were assigned and received ≥ 1 dose of the study drug. The FAS was used for all efficacy analyses except NEDA-3, which used a modified FAS excluding participants who achieved NEDA-3 but then discontinued treatment prematurely for reasons other than lack of efficacy or death. ARR was analyzed using a negative binomial regression model with log-link and adjusted for prior MS therapies (fingolimod or fumarates), number of relapses in previous year, and participant’s baseline EDSS, number of T1 Gd + lesions, and age as covariates. The natural log of the time-in-study (in years) was used as offset. Safety analyses included the Safety Set, which included all participants who received ≥ 1 dose of study drug, and used data collected through 100 days after the last study drug administration or permanent treatment discontinuation. TEAEs (new or worsening from baseline) are summarized by system organ class and/or preferred term, severity (based on the most current Common Terminology Criteria for Adverse Events grading system), adverse event type, and relation to study drug. For exploratory endpoints, the number of T1 Gd + lesions per scan and the number of neT2 lesions per year were analyzed using a negative binomial regression model, while participants meeting NEDA-3 and 6mCDW were reported descriptively and via Kaplan–Meier estimate, respectively. Statistical hypotheses were tested at the 5% significance level, and *p* values less than 0.05 were considered statistically significant for the primary endpoint.

## Results

### Patient disposition, baseline demographics, and clinical characteristics

A total of 562 participants who had breakthrough disease on fingolimod (n = 181; 32.2%) or fumarates (n = 381; 67.8%) were enrolled. Of these, 39 participants (6.9%) discontinued the study, primarily due to participant decision (n = 24); 523 participants (93.1%) completed the 96 weeks of treatment with ofatumumab (Online Resource Fig. 1). Mean ofatumumab exposure was 91.8 weeks.

Participants switching from fingolimod had more advanced MS at baseline compared with participants switching from fumarates (Table 1). This was evidenced by longer MS disease duration since diagnosis, a greater number of relapses in the last 12–24 months prior to screening, a lower proportion of participants free of Gd + T1 lesions, higher T2 lesion volume, lower SDMT scores, and treatment with a higher number of prior DMTs in the fingolimod group compared with participants switching from fumarates (all p < 0.05; Table 1).

**Table 1:** Baseline demographics and clinical characteristics (FAS): overall and by prior DMT

Characteristic	Overall population (N = 562)	By last prior DMT	
		Fingolimod (n = 181)	Fumarates (n = 381)
Age, years	36.3 ± 9.65	36.1 ± 9.71	36.4 ± 9.63
Female, n (%)	369 (65.7)	121 (66.9)	248 (65.1)
Male, n (%)	193 (34.3)	60 (33.1)	133 (34.9)
EDSS	2.32 ± 1.143	2.40 ± 1.154	2.29 ± 1.137
SDMT, number of correct answers in 90 seconds^a^	52.8 ± 13.87^b^	51.1 ± 14.40^a^	53.6 ± 13.56^a,c^
Proportion of participants free of Gd + T1 lesions, n (%)^a^	401 (71.4)	113 (62.4)^a^	288 (75.6)^a^
Number of Gd + T1 lesions	1.3 ± 4.96^d^	1.9 ± 5.39^e^	1.0 ± 4.72^f^
Total volume of T2 lesions, cm^3^	10.56 ± 11.68^ g^	12.47 ± 12.30^a^	9.65 ± 11.28^a,h^
Number of prior DMTs, n (%)^a^ 1 2 3 4	269 (47.9) 207 (36.8) 85 (15.1) 1 (0.2)	61 (33.7)^a^ 82 (45.3)^a^ 37 (20.4)^a^ 1 (0.6)^a^	208 (54.6)^a^ 125 (32.8)^a^ 48 (12.6)^a^ 0^a^
Duration of MS since diagnosis, years^a^	5.42 ± 2.81	6.06 ± 2.72^a^	5.11 ± 2.81^a^
Number of relapses in the last 12–24 months prior to screening	0.8 ± 1.04	1.1 ± 1.29^a^	0.8 ± 0.89^a^
IgG, g/L^a^	9.97 ± 2.05^ g^	9.10 ± 1.95^a^	10.38 ± 1.97^a,h^
IgM, g/L^a^	1.16 ± 0.64^ g^	0.99 ± 0.57^a^	1.24 ± 0.66^a,h^
Lymphocytes (10^9^/L)^a^	1.36 ± 0.55^ g^	1.12 ± 0.48^a,i^	1.48 ± 0.54^a^
Duration of washout, days, n (%)^a^ < 30 days ≥ 30 days	251 (44.7) 311 (55.3)	47 (26.0)^a^ 134 (74.0)^a^	204 (53.5)^a^ 177 (46.5)^a^

Unless specified otherwise, values are represented as mean ± SD. One participant with a history of 4 prior DMTs was included in both the FAS and SF; this was documented as a protocol deviation

^a^ p < 0.05 between the fingolimod and fumarate subgroups

Number of participants with available data at baseline: ^b^ n = 560. ^c^ n = 379. ^d^ n = 556. ^e^ n = 178. ^f^ n = 378. ^g^ n = 561. ^h^ n = 380. ^i^ n = 180

*DMT* disease-modifying therapy; *EDSS* Expanded Disability Status Scale; *FAS* full analysis set; *Gd +* gadolinium enhancing; *Ig* immunoglobulin; *MS* multiple sclerosis; *SF* safety set; *SD* standard deviation; *SDMT* symbol digit modalities test; *SF* safety set

### Efficacy

#### Annualized relapse rate (primary endpoint)

Over 96 weeks, the adjusted ARR in the overall population was low at 0.06 (95% CI: 0.05–0.08; p < 0.0001), meeting the threshold for significance. The adjusted ARR was 0.09 for participants transitioning from fingolimod (95% CI: 0.06–0.13) and 0.06 (95% CI: 0.04–0.08) for those transitioning from fumarates. There was a notable decrease in the adjusted ARR from Year 1 (0.10; 95% CI: 0.07–0.13) to Year 2 (0.02; 95% CI: 0.01–0.04).

#### MRI-associated endpoints

**Fig. 2:**
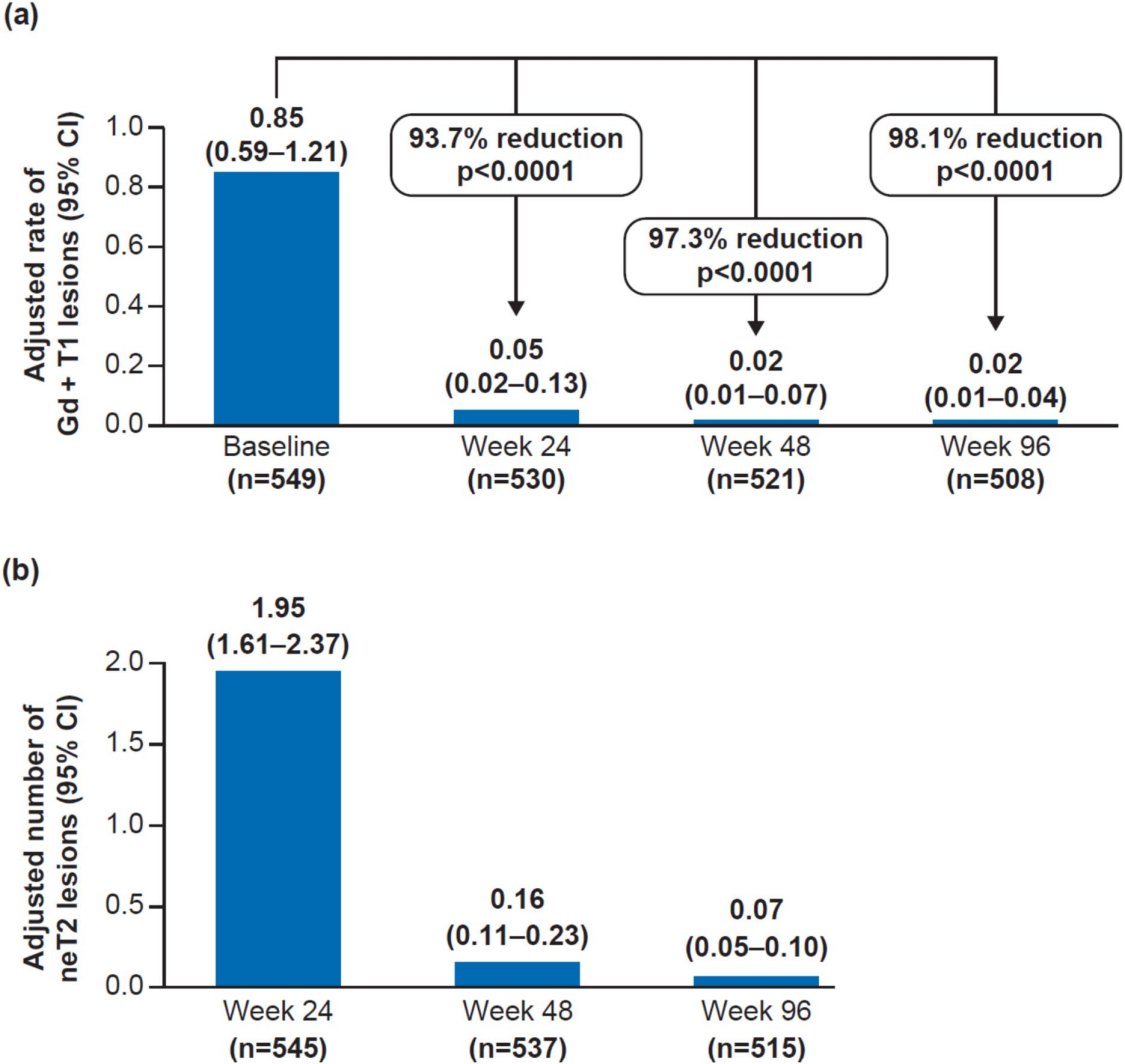
MRI activity in the overall population over 96 weeks following the switch from fingolimod or fumarates to ofatumumab**. a** Adjusted rate of Gd + T1 lesions per scan, estimated using a negative binomial regression model with a log link, adjusted for prior MS therapy (fingolimod or fumarates) and visit as factors, and for number of relapses in the previous year, baseline EDSS score, and age at baseline as covariates. The natural log of the number of scans was used as an offset for Gd + lesions; **b** Annualized rate of new or enlarging T2 lesions, estimated using a negative binomial regression model with a log link, adjusted for prior MS therapy and visit as factors, and for number of relapses in the previous year, baseline EDSS score, age at baseline, and baseline T2 lesion volume as covariates. The natural log of time since the previous MRI scan (in years) was used as an offset. *EDSS* Expanded Disability Status Scale; *Gd*+ gadolinium-enhancing; *MRI* magnetic resonance imaging; *MS* multiple sclerosis; *neT2* new or enlarging T2

Ofatumumab treatment significantly reduced the adjusted rate of Gd + T1 lesions per scan at Week 24 (0.05; 95% CI: 0.02–0.13; p < 0.0001) compared with baseline (0.85; 95% CI: 0.59–1.21). Gd + T1 lesions were almost completely suppressed at Weeks 48 (0.02; 95% CI: 0.01–0.07; p < 0.0001) and 96 (0.02; 95% CI: 0.01–0.04; p < 0.0001); representing reductions in detected Gd + T1 lesions of 93.7%, 97.3%, and 98.1% at Weeks 24, 48, and 96, respectively (Fig. 2a). At baseline, the proportion of participants in the fumarate subgroup with no Gd + T1 lesions was higher than in the fingolimod subgroup (76.2% versus 64.0%); however, the proportion of participants free of Gd + T1 became similar in both subgroups by Week 24 and beyond following the switch to ofatumumab, despite initial differences at baseline (Online Resource Table 2).

Participants’ neT2 lesions were almost completely suppressed between Weeks 24 and 96 in the overall population and by prior DMT subgroup. At Week 24, the adjusted number of neT2 lesions was 1.95 (95% CI: 1.61–2.37), decreasing to 0.16 (95% CI: 0.11–0.23) at Week 48, and 0.07 (95% CI: 0.05–0.10) at Week 96 (Fig. 2b). The decreases in the annualized neT2 lesion rate were accompanied by decreases in lesion load, as shown by decreases from baseline in T2 lesion volume (Online Resource Fig. 2**).**

#### NEDA-3

**Fig. 3:**
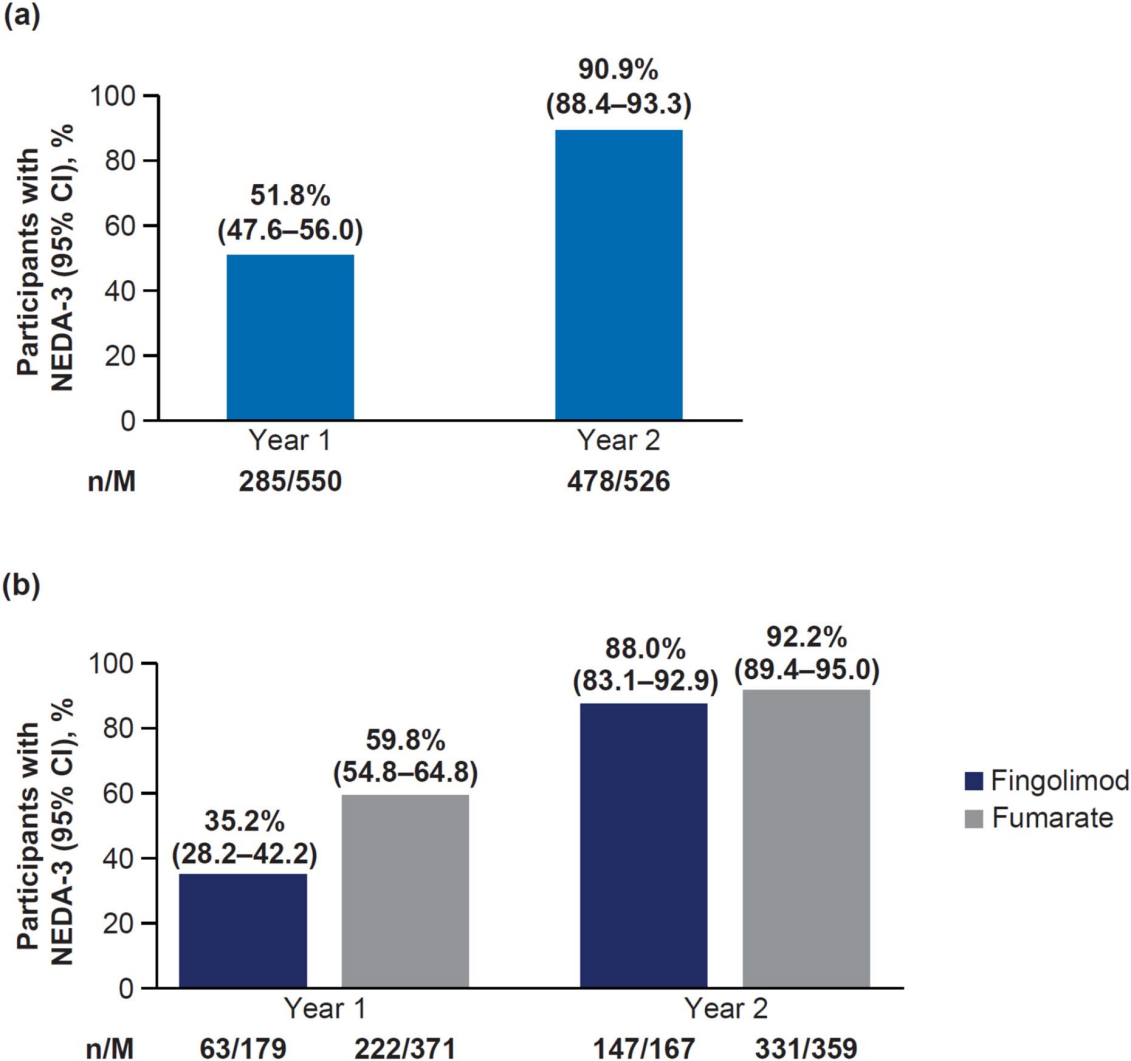
Proportion of participants achieving NEDA-3 in Years 1 and 2 following switch to ofatumumab treatment, measured overall and by prior DMT. **a** NEDA-3 by year in the overall population; and **b** NEDA-3 by prior DMT subgroup by year, where NEDA-3 was defined as no 6mCDW, no confirmed MS relapse, no new or enlarging T2 lesions compared with baseline, and no gadolinium-enhancing (Gd +) T1 lesions. The modified FAS for NEDA-3 included all participants in the FAS, but participants who discontinued from the study treatment prematurely for reasons other than lack of efficacy or death and achieved NEDA-3 before treatment discontinuations were excluded. *6mCDW* 6-month confirmed disability worsening; *DMT* disease-modifying therapy; *FAS* full analysis set; *M* total number of participants in the treatment group with response variable defined; *n* number of participants achieving NEDA-3; *NEDA* no evidence of disease activity

In the overall population, NEDA-3 was achieved by 51.8% of participants in Year 1 (95% CI: 47.6–56.0) and 90.9% in Year 2 (95% CI: 88.4–93.3) (Fig. 3a). When stratified by prior DMT (Fig. 3b), in the fingolimod subgroup, 35.2% achieved NEDA-3 in Year 1 (95% CI: 28.2–42.2) and 88.0% in Year 2 (95% CI: 83.1–92.9). In the fumarate subgroup, 59.8% achieved NEDA-3 in Year 1 (95% CI: 54.8–64.8) and 92.2% in Year 2 (95% CI: 89.4–95.0).

#### 6mCDW

**Fig. 4:**
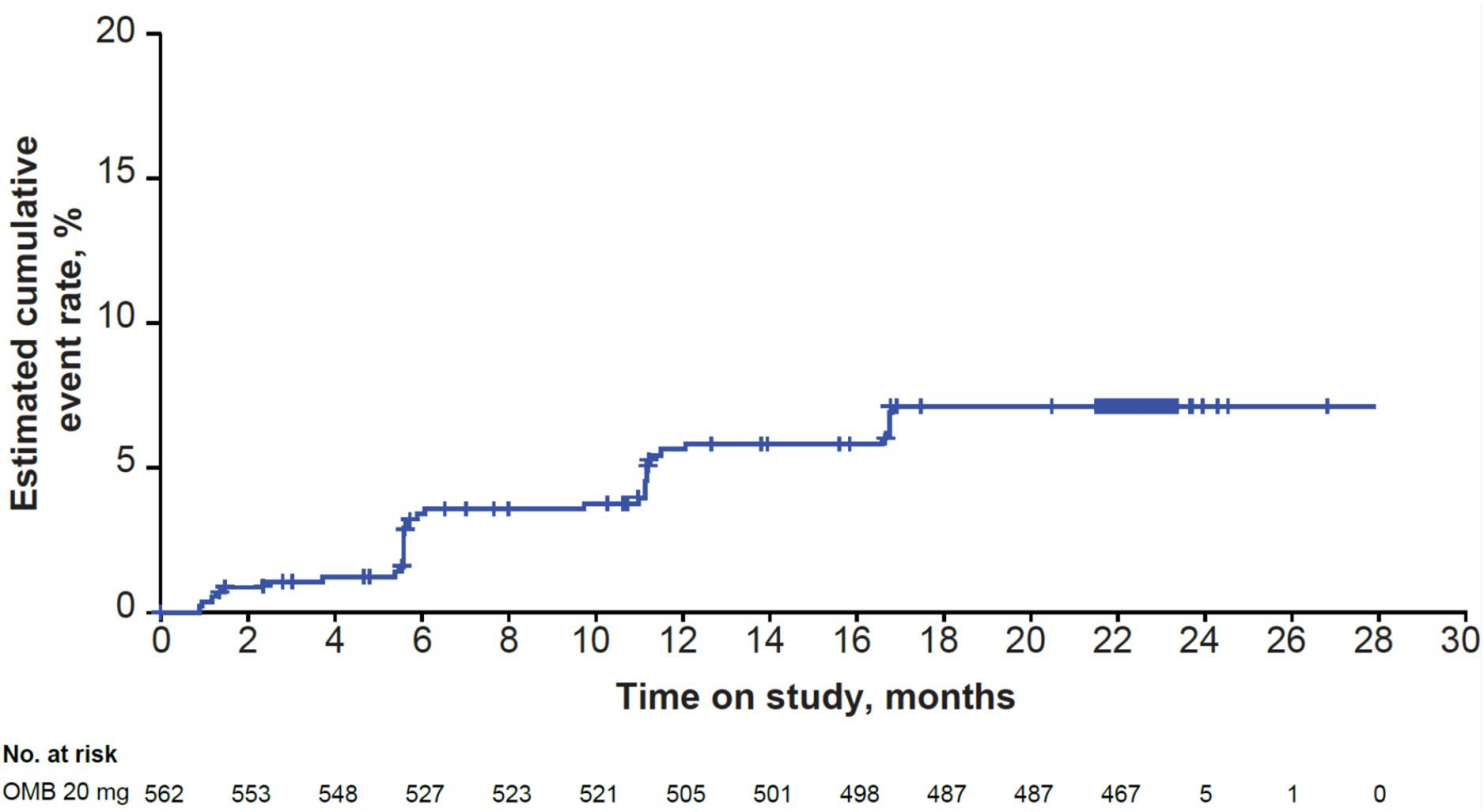
Number of 6mCDW events over the 96-week treatment period in the overall population. *6mCDW* 6-month confirmed disability worsening; *KM* Kaplan–Meier; *OMB* ofatumumab

During the 96-week treatment period, 40 participants experienced 6mCDW (7.3%; 95% CI: 5.4–9.9) (Fig. 4), including 15 who switched from fingolimod (8.5%; 95% CI: 5.2–13.8) and 25 who switched from fumarates (6.8%; 95% CI: 4.6–9.9).

#### Additional exploratory endpoints and PROs

No deterioration in function was identified across exploratory endpoints and PROs. Scores for the T25FW, 9HPT, SDMT, and LCVA remained stable, with changes from baseline not reaching clinically meaningful thresholds (Online Resource Figs. 3–6). For the 6mCCD, the cumulative event rates were 11.4% and 11.9% at Year 1 and Year 2, respectively (Online Resource Table 3). NfL concentrations decreased from Week 24 and remained stable through 96 weeks (Online Resource Fig. 7).

Among the PROs assessed, TSQM scores exhibited gradual improvements over the course of the study, as illustrated by higher overall patient satisfaction, treatment effectiveness, and treatment convenience (Online Resource Fig. 8). MSIS-29 physical and psychological domain scores remained stable, with no indication of functional decline (Online Resource Fig. 9). Similarly, changes versus baseline in fatigue scores per the FSMC did not show deterioration, with no clinically meaningful changes observed through the treatment period (Online Resource Table 4).

#### Safety

TEAEs and TEAEs stratified by last prior DMT are shown in Table 2. Overall, 509 participants (90.6%) reported a TEAE, with similar percentages between those switching from fingolimod (90.1%) or fumarates (90.8%). Most TEAEs (90.6%) were mild or moderate (grade 1 or 2) in severity. Infections and infestations were the most frequently reported system organ class (SOC), with TEAEs reported in 69.8% of participants.

**Table 2:** Safety profile in participants receiving ofatumumab: overall and by prior DMT

All grades, n (%)	Overall population (N = 562)	By last prior DMT	
		Fingolimod (n = 181)	Fumarates (n = 381)
Participants with ≥ 1 AE	509 (90.6)^a^	163 (90.1)	346 (90.8)
Participants with ≥ 1 SAE	33 (5.9)	13 (7.2)	20 (5.2)
Participants with drug-related AE(s)	374 (66.5)	115 (63.5)	259 (68.0)
Participants with AE(s) causing study drug discontinuations	5 (0.9)^b^	3 (1.7)	2 (0.5)
Participants with AE(s) causing study drug interruptions	33 (5.9)^c^	11 (6.1)	22 (5.8)

^a^ A total of 41 (7.3%) participants had grade 3 or 4 AEs

^b^ Reasons for treatment discontinuation include COVID-19 (n = 1), abnormal MRI (consisting of a lesion initially suspicious for progressive multifocal leukoencephalopathy, later confirmed as new MS lesions, that led to study discontinuation based on investigator judgment; n = 1), and neoplasms (benign, malignant, and unspecified [including cysts and polyps]) (n = 3)

^c^ Drug interruptions were caused by gastrointestinal disorders (n = 3), infections and infestations (n = 32; COVID-19 [n = 23]), UTI [n = 2], other infections and infestations [n = 9]), migraine (n = 1) and suicidal ideation (n = 1). Participants could have discontinued or interrupted drug for ≥ 1 AE

*AE* adverse event; *DMT* disease-modifying therapy; *MRI* magnetic resonance imaging; *SAE* serious adverse event; *UTI* urinary tract infection

TEAEs by preferred term that occurred in ≥ 10% of participants are listed in Online Resource Table 5. The most common TEAEs were systemic IRRs (53.4%), followed by COVID-19 (37.0%), nasopharyngitis (17.1%) and headache (16.4%). COVID-19 cases were predominantly mild or moderate (99.5%), with 1 case classified as grade 3. Serious TEAEs were reported in 33 participants (5.9%), with 13 (7.2%) switching from fingolimod and 20 (5.2%) switching from fumarates (Online Resource Table 6). Three serious TEAEs were reported by > 1 participant: uterine leiomyoma, suicidal ideation, and intervertebral disc protrusion were reported in 2 participants (0.4%) each. No deaths were reported during the study or the subsequent safety assessment period.

TEAEs resulting in treatment interruption were reported in 33 participants (5.9%) (Online Resource Table 7), largely arising from COVID-19 (23 [4.1%]). Five participants (0.9%) discontinued ofatumumab treatment due to TEAEs, including COVID-19 (n = 1), abnormal MRI (consisting of a lesion initially suspicious for progressive multifocal leukoencephalopathy, later confirmed as new MS lesions, that led to study discontinuation based on investigator judgment; n = 1), and cancer (n = 3). Cancer cases included adenocarcinoma (n = 1), bladder cancer (n = 1), and medullary carcinoma of breast (n = 1).

#### Injection-related reactions

**Fig. 5:**
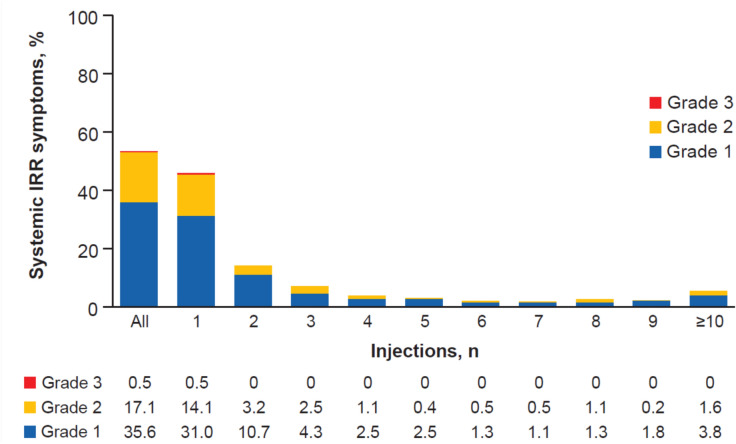
Overall incidence of systemic injection-related reactions by injection number and severity. The injection number reflects the sequential number of actual injections received. M is the number of participants with the specified injections (% = n/M × 100). A participant with multiple events of the same symptom is counted only once per injection. *IRR* injection-related reaction; *M* total number of participants with the specified injections; *n* number of participants with any symptom

A breakdown of systemic IRRs is detailed in Fig. 5. Local-site IRRs were reported in 62 participants (11.0%). Nearly all systemic IRRs (296 of 299) were mild to moderate in severity, with grade 1 systemic IRRs reported in 200 participants (35.6%), grade 2 in 96 participants (17.1%), and grade 3 in 3 participants (0.5%). The incidence of systemic IRRs decreased substantially after the first injection (45.6% following the first injection versus a cumulative 7.6% across all subsequent injections; Fig. 5). All local-site IRRs were mild to moderate in severity (grade 1 or 2). No grade 4 or serious IRR events were reported. The most common symptom associated with systemic IRRs was fever, followed by chills and headache. No IRRs led to treatment interruption or discontinuation.

#### IgG/IgM levels

Mean serum IgG levels remained stable and above the lower limit of normal (LLN; 5.65 g/mL) through Week 96 for the overall study population and in both prior DMT subgroups (Online Resource Fig. 10). Mean serum IgM levels in the overall study population and prior DMT subgroups decreased from baseline to Week 96 but remained above the LLN (0.4 g/L) at all time points. A low number of participants had ≥ 1 Ig level below LLN post baseline (2.1% for IgG and 22.8% for IgM); these events were not associated with severe infections or treatment discontinuations.

#### Post hoc efficacy and safety analyses

Post hoc efficacy analysis revealed no clinically meaningful differences in efficacy endpoints when analyzed by washout duration before switching to ofatumumab (Online Resource Figs. 11–15). Rebaselining efficacy endpoints at Month 6 resulted in outcomes similar to those in the original dataset (Online Resource Tables 8–11), although the proportion of participants experiencing 6mCDW decreased following rebaselining (Fig. 4). Safety outcomes were generally consistent across washout durations, with types of TEAEs occurring in ≥ 10% of participants being comparable across subgroups (Online Resource Table 12).

## Discussion

ARTIOS demonstrated that people with RMS switching to ofatumumab after breakthrough disease on fingolimod or fumarates had a substantial reduction in relapses and MRI activity, and few 6mCDW events.

Initial baseline characteristics indicated more advanced disease among participants switching from fingolimod than in those switching from fumarates. Despite these differences, ofatumumab treatment demonstrated consistent and comparable efficacy levels across both prior DMT subgroups.

Ofatumumab treatment resulted in a low ARR (0.06) over 96 weeks in the overall population, and in each prior DMT subgroup (fingolimod, 0.09; fumarates, 0.06). Overall, the low ARRs observed with ofatumumab in ARTIOS are consistent with those seen in participants treated with ofatumumab in the ASCLEPIOS trials [14]. The switch to ofatumumab also resulted in a substantial reduction of MRI activity in the overall population and by prior DMT. Ofatumumab treatment significantly reduced the rate of Gd + T1 lesions at Week 24 versus baseline, and Gd + T1 lesions were almost completely suppressed at Weeks 48 and 96. Similarly, ofatumumab almost completely suppressed neT2 lesions at Week 48 and 96 compared with Week 24, and reductions in MRI lesion load versus baseline were seen at Weeks 48 and 96. Overall, the ARR and MRI results support the strong anti-inflammatory effects of ofatumumab in participants with RMS and, particularly, in participants with breakthrough disease despite treatment with oral fingolimod or fumarates.

The low percentage (7.3%) of participants with 6mCDW events over the 2-year study duration further supports the clinical impact of ofatumumab treatment, consistent with confirmed disability worsening event rates seen with other anti-CD20 agents [20].

The low rates of ARR, near-complete suppression of MRI activity, and few 6mCDW events led to high NEDA-3 rates, with > 9 of every 10 participants (90.9%) experiencing NEDA-3 in Year 2, with similar outcomes observed in both prior DMT subgroups despite the initial difference in baseline disease activity. Achieving NEDA-3 during the first 2 years of treatment has been associated with a lower risk of long-term disability [21, 22], further supporting the switch to HETs among participants experiencing suboptimal responses to fingolimod or fumarates.

Concentrations of NfL, a neuron-specific protein that is a marker of neuro-axonal damage in neurological disease, decreased from Week 24 following the switch to ofatumumab and then remained stable through 96 weeks [23–25]. The reduction and stabilization of serum NfL levels observed following the switch to ofatumumab are consistent with improved MS disease control and with observed ARR and MRI improvements seen in the current study.

Additional exploratory efficacy assessments, including assessments for ambulation (T25FW), upper limb function (9HPT), cognitive processing speed (SDMT), and visual acuity (LCVA) indicated stability in disease measures over time, with all endpoints remaining functionally stable. Overall, 11.4% and 11.9% of participants experienced 6mCCD in Years 1 and 2, respectively, supporting an apparent stabilization of cognitive decline in Year 2 of ofatumumab treatment.

Regarding PRO assessments, MSIS-29 and FSMC scores remained stable over the course of study, while TSQM scores improved, indicating higher patient satisfaction, effectiveness, and convenience after switching to ofatumumab [26]. This result may be a positive indication for strong future adherence [27].

Recurring disease activity is a known risk factor for participants discontinuing fingolimod therapy [16, 17]. While no notable evidence of rebound was observed in this study (as demonstrated by the similarity in efficacy endpoints when analyzed by washout duration), participants switching from fingolimod after a 30-to-60–day washout showed a small, nonsignificant increase in Gd + T1 lesions and neT2 lesions compared with those who had a washout duration of < 30 days. Increases of a similar extent were not observed in participants with washout durations ≥ 60 days (possibly due to rebound activity occurring prior to the screening period), and clinically meaningful improvements in efficacy outcomes (ARR, MRI lesions, and NEDA-3) were not observed in the Month 6 rebaselined analyses. While further data is needed for more definitive conclusions, these preliminary findings may help inform clinicians of the potential advantages of a shorter (< 30-day) washout in preventing rebound potential. Additionally, no safety concerns were identified that would preclude a shorter washout duration.

The overall safety profile of ofatumumab in the ARTIOS study was consistent with previous findings [28–30]. Most TEAEs (90.6%) were mild to moderate, with low rates of serious TEAEs and treatment discontinuations or interruptions. Overall rates of TEAEs and serious TEAEs in ARTIOS were similar to those reported in the pivotal ASCLEPIOS trials [14]. In ARTIOS, the most frequent TEAEs by SOC were infections and infestations; however, the study was initiated during the first year of the COVID-19 pandemic, which may have impacted this finding. Excluding COVID-19 cases, the infection rates were lower than in the ASCLEPIOS trials [14]. In ARTIOS, most COVID-19 cases were mild or moderate, with only 1 case classified as grade 3. These findings are noteworthy and highlight a safety profile of ofatumumab differentiated from that of other anti-CD20 agents, as prior studies reported an increased risk of severe COVID-19 outcomes among people with MS treated with certain anti-CD20 therapies [31, 32].

While a higher rate of IRRs was observed in ARTIOS compared with ASCLEPIOS [14], most IRRs were mild to moderate, with few Grade 3 IRRs reported. Most IRRs occurred after the first injection, and none led to treatment discontinuation or interruption. Notably, premedication use was lower in ARTIOS compared with ASCLEPIOS, which may have contributed to the higher incidence of IRRs reported in ARTIOS [14]. Mean IgG and IgM levels were also consistent with previously reported findings for ofatumumab, with mean serum IgG levels remaining stable and above the LLN (5.65 g/L) for ≤ 2 years, and mean serum IgM levels decreasing from baseline to Week 96 but remaining above the LLN (0.4 g/L). Of note, long-term data from the ALITHIOS open-label extension study demonstrated that mean IgG levels remained stable and above the LLN with continued ofatumumab treatment [29, 33]. Sustained Ig levels may differentiate ofatumumab from other anti-CD20 therapies and could be relevant to the favorable COVID-19 outcomes observed in participants treated with ofatumumab [14]. Although IgG and IgM levels were lower at baseline in the fingolimod subgroup compared with the fumarate subgroup, no differences in IgG or IgM levels were observed following the switch to ofatumumab, in line with prior long-term data [29].

Limitations of the study include the single-arm design, which limits the ability to draw definitive conclusions regarding efficacy or to compare outcomes in patients switching from fingolimod or fumarates to a different HET, as well as the short follow-up period. In addition, as participants were enrolled during periods of breakthrough disease activity, the potential for outcomes to be partly due to “regression to the mean” should be considered. Lastly, due to the low number of participants aged > 55 years and the short study duration, the ability to assess potential impact of older age on NfL levels may have been limited, which should be considered when interpreting results for this exploratory endpoint. Strengths include the assessment of a patient population often encountered in clinical practice (participants with breakthrough disease despite receiving oral DMTs) who were underrepresented in the phase 3 ASCLEPIOS studies, the inclusion of several exploratory endpoints and PROs, and the study design, which allowed alignment with local labels and provided investigators flexibility in assessment to more closely reflect a real-world environment.

## Conclusions

Data from the ARTIOS study supports ofatumumab as a HET with a favorable safety profile in participants with breakthrough disease on oral fingolimod or fumarates. Switching to ofatumumab resulted in strong disease control, with consistent efficacy outcomes across prior DMT subgroups, including in participants who switched from fingolimod, despite these participants having more advanced disease at baseline. No new safety concerns were observed, and safety findings were similar across prior DMT subgroups and consistent with findings of previous ofatumumab trials [14]. Treatment satisfaction strengthened during the study. Overall, results from ARTIOS complement the pivotal phase 3 ASCLEPIOS studies and support the efficacy and safety of ofatumumab following switch from oral DMTs in people with RMS with suboptimal responses to fingolimod or fumarates.

## Supplementary Information

Below is the link to the electronic supplementary material.

Supplementary file1 (DOCX 718 KB)

## Data Availability

Novartis is committed to sharing with qualified external researchers, access to patient-level data and supporting clinical documents from eligible studies. These requests are reviewed and approved by an independent review panel on the basis of scientific merit. All data provided are anonymized to respect the privacy of patients who have participated in the trial in line with applicable laws and regulations. The trial data availability is according to the criteria and process described on http://www.clinicalstudydatarequest.com.
