# Supplementary material for: Efficacy and safety of ofatumumab in participants with relapsing multiple sclerosis and breakthrough disease on oral fingolimod or fumarates: results from the ARTIOS study
Source: J Neurol. 2026 Jun 29;273(7):434. doi: 10.1007/s00415-026-13960-5 (PMC13315151; doi:10.1007/s00415-026-13960-5)
Supplement: Supplementary file 1 — Supplementary file1 (DOCX 718 KB) [file 415_2026_13960_MOESM1_ESM.docx]

**SUPPLEMENTARY INFORMATION**

Efficacy and Safety of Ofatumumab in Participants With Relapsing Multiple Sclerosis and Breakthrough Disease on Oral Fingolimod or Fumarates: Results From the ARTIOS Study

**Authors:** Riley Bove^1^, Dawn Langdon^2^, Maciej Maciejowski^3^, Elzbieta Jasinska^4^, Michael Dufek^5^, Anil Abeyewickreme^6^, Anamaria Rauh^7^, Haoyi Fu^8^, Imran Ali Khan^9^, Matthias Boehringer^10^, Sara Eichau Madueno^11^, Tobias Derfuss^12^

^1^University of California San Francisco, San Francisco, CA, USA

^2^Royal Holloway, University of London, London, UK

^3^MA-LEK, MS Center, Katowice, Poland

^4^Collegium Medicum, Jan Kochanowski University, Kielce, Poland

^5^First Department of Neurology, St. Anne’s University Hospital, Masaryk University, Brno, Czechia

^6^Novartis Pharmaceuticals UK, London, UK

^7^Novartis Pharma AG, Basel, Switzerland

^8^Novartis Pharmaceuticals Corporation, East Hanover, NJ, USA

^9^Novartis Healthcare Private Limited, Hyderabad, India

^10^Neurozentrum Bielefeld, Bielefeld, Germany

^11^Department of Neurology, Hospital Universitario Virgen Macarena, Seville, Spain ^12^Neurology Clinic and Policlinic and Research Center for Clinical Neuroimmunology and Neuroscience, Departments of Medicine and Biomedicine, University Hospital and University of Basel, Basel, Switzerland

**Corresponding Author**

Correspondence to Tobias Derfuss: [Tobias.Derfuss@usb.ch](mailto:Tobias.Derfuss@usb.ch)

**Multiple sclerosis (MS) relapse definition**

MS relapse, assessed at all study visits, was defined as the appearance of a new neurological abnormality or worsening of previously stable or improving pre-existing neurological abnormality, separated by ≥30 days from onset of a preceding clinical demyelinating event. The abnormality must have been present for ≥24 hours and occur in the absence of fever (<37.5°C) or known infection. A confirmed relapse was defined as a relapse accompanied by a clinically relevant change in Expanded Disability Status Scale (EDSS; ie, increase of ≥0.5 points on the EDSS score), or an increase of 1 point on two functional scores (FS) or 2 points on one FS, excluding changes involving bowel, bladder, or cerebral FS compared with the previous available rating (ie, last EDSS rating that did not occur during a relapse).

**Inclusion criteria**

Participants were eligible for the study if they met the following criteria:

- Age 18-60 years at screening
- A diagnosis of MS (based on the revised criteria from McDonald 2017 [19]
- Diagnosis of relapsing forms of MS (RMS), including secondary progressive MS (SPMS)
- An EDSS score of 0 to 4 at the time of screening
- Received fingolimod or fumarates as their most recent disease modifying therapy (DMT) administered for ≥6 months prior to transition to the study drug. Participants transitioning from a prior DMT were grouped such that all fumarate-based therapies (eg, dimethyl fumarate [DMF] and diroximel fumarate [DRF]) were considered as a single DMT
- Experienced breakthrough disease activity while using an adequate regimen of fingolimod or fumarates, as evidenced by ≥1 clinically reported relapses or ≥1 signs of MRI activity (eg, gadolinium-enhancing [Gd+] or new or enlarging T2 lesions [neT2])
- Neurologically stable during the 1 month preceding the first administration of the study drug

**Exclusion criteria**

Participants meeting any of the following criteria were not eligible for inclusion:

- Being unable or unwilling to cooperate or comply with study protocol requirements in the opinion of the investigator
- Having primary progressive MS or SPMS without disease activity
- Meeting criteria for neuromyelitis optica
- Disease duration of >10 years since diagnosis
- Pregnant or nursing (lactating) women, confirmed by a positive human chorionic gonadotropin laboratory test
- Women of childbearing potential, defined as all women physiologically capable of becoming pregnant, unless they were using highly effective methods of contraception during dosing and for ≥6 months after stopping study medication. Highly effective contraception methods included:
  - Total abstinence (when this was in line with the preferred and usual lifestyle of the participant, if accepted by the local regulation). Note: Periodic abstinence (eg, calendar, ovulation, symptothermal, or postovulation methods) and withdrawal were not acceptable methods of contraception
  - Female bilateral tubal ligation, female sterilization (had surgical bilateral oophorectomy with or without hysterectomy) or total hysterectomy ≥6 weeks before taking study treatment. In case of oophorectomy alone, only when the reproductive status of the woman was confirmed by follow-up hormone level assessment
  - Male partner sterilization (≥6 months prior to screening). For female participants on the study, the vasectomized male partner should have been the sole partner
  - Use of oral (estrogen and progesterone), injected, or implanted hormonal methods of contraception or placement of an intrauterine device (IUD), intrauterine system (IUS), or other forms of hormonal contraception that have comparable efficacy (failure rate <1%), eg, hormone vaginal ring or transdermal hormone contraception. In the case of use of oral contraception, women should have been stable on the same pill for a minimum of 3 months before taking study treatment
  - If local regulations were more stringent than the contraception methods listed above, local regulations applied and are described in the informed consent form
  - Women were considered postmenopausal if they had 12 months of natural (spontaneous) amenorrhea with an appropriate clinical profile (eg, age-appropriate history of vasomotor symptoms). Women were considered not of childbearing potential if they were postmenopausal or had surgical bilateral oophorectomy (with or without hysterectomy), total hysterectomy, or bilateral tubal ligation ≥6 weeks prior to enrollment on study. In the case of oophorectomy alone, the woman was considered not to be of child-bearing potential only after reproductive status was confirmed by follow-up hormone level assessment
- Active chronic disease (or stable but treated with immune therapy) of the immune system other than MS (eg, rheumatoid arthritis, scleroderma, Sjögren syndrome, Crohn disease, ulcerative colitis, etc) or with immunodeficiency syndrome (hereditary or drug-induced immune deficiency)
- Active systemic bacterial, fungal, or viral infection (such as HIV or COVID-19). Where local regulation required it, SARS-CoV-2 must have been ruled out by the polymerase chain reaction (PCR) test
- Neurological symptoms consistent with progressive multifocal leukoencephalopathy (PML) or confirmed PML
- Participants at risk of developing or having reactivation of syphilis or tuberculosis (eg, those with known exposure to or history of syphilis or active or latent tuberculosis, even if previously treated), as confirmed by medical history or per local practice
- Participants with active hepatitis B or C disease, assessed locally
  - Hepatitis B virus (HBV) screening was to be performed before initiation of treatment. At a minimum, screening included hepatitis B surface antigen (HBsAg) and hepatitis B core antibody (anti-HBc) testing. These could be complemented with other appropriate markers as per local guidelines. Participants with positive hepatitis B serology (either HBsAg or anti-HBc) should have an HBV DNA test performed. If HBV DNA test was positive, the participant was not eligible. If HBV DNA test was negative, the participant needed to consult a liver disease expert before the start of treatment and be monitored and managed following local medical standards to prevent hepatitis B reactivation
  - Hepatitis C virus (HCV) risk must have been ruled out via anti-HC immunoglobulin G (IgG; if positive on IgG, HCV-RNA PCR was performed; if negative, the participant could be enrolled). Note: If the investigator suspected false-positive hepatitis serology results, such as an antibody pattern indicating acute hepatitis infection but no corresponding elevated liver enzymes and no signs or symptoms of liver disease, an infectious disease expert could be consulted. If the potential participant had a record of vaccination including HB, and there was no evidence of acute or chronic hepatitis infection (confirmed by an infectious disease expert), the investigator must have documented (in source data and as a comment in the electronic case report form) that the serology results were considered false positive and may then have enrolled the participant
- Have received any live or live-attenuated vaccines (including for *Varicella zoster* virus or measles) ≤4 weeks prior to first study drug administration
- Have been treated with any of the medications listed below within the time specified:
  - Systemic corticosteroids or adrenocorticotropic hormone ≤30 days prior to screening MRI scan
  - Teriflunomide (unless rapid elimination procedure was performed) ≤9 months prior to first study drug administration
  - Natalizumab ≤6 months prior to first study drug administration
  - Highly immunosuppressive/chemotherapeutic medications (eg, mitoxantrone, cyclophosphamide, cladribine, daclizumab, B-cell targeted therapies such as rituximab and ocrelizumab, laquinimod) ≤2 years prior to first study drug administration
  - At any time: mitoxantrone (with evidence of cardiotoxicity following treatment or cumulative lifetime dose >60 mg/m^2^), alemtuzumab, lymphoid irradiation, bone marrow transplant, other strongly immunosuppressive treatments (with effects potentially lasting >6 months), ofatumumab, any aCD20+ monoclonal antibody in development (eg, ublituximab or obinutuzumab)
- Use of other investigational drugs at enrollment (screening) or within the prior 30 days, or 5 elimination half-lives, or until the expected pharmacodynamics effect has returned to baseline, whichever is longer
- History of malignancy of any organ system (other than basal cell carcinoma, in situ squamous cell carcinoma of skin, or in situ carcinoma of cervix or the uterus that has been radically treated, eg, completely excised with clear margins) within the past 5 years, regardless of whether there is evidence of local recurrence or metastases
- Any of the following chronic, severe conditions or treatments that may impact the

compliance of the study:

- - History of, or current, significant cardiac disease, including cardiac failure (New York Heart Association functional class II-IV), myocardial infarction (≤6 months prior to screening), unstable angina (≤6 months prior to screening), transient ischemic attack (≤6 months prior to screening), stroke, cardiac arrhythmias requiring treatment or uncontrolled arterial hypertension
  - Concomitant clinically significant cardiac arrhythmias, eg sustained ventricular tachycardia and clinically significant second- or third-degree atrioventricular block without a pacemaker on screening electrocardiogram
  - History of active severe respiratory disease, including chronic obstructive pulmonary disease, interstitial lung disease, or pulmonary fibrosis
  - Asthma requiring regular treatment with oral steroids
  - Severe hepatic impairment (Child-Pugh class C) or any chronic liver or biliary disease
  - Severe renal impairment (glomerular filtration rate <30 mL/min/1.73 m^2^)
  - Any medically unstable condition as determined by the Investigator
  - Severe hypoproteinemia, eg, in nephrotic syndrome
- Any of the following abnormal laboratory values as confirmed by the central laboratory prior to first study drug administration:
  - Lymphocyte count <500/mm^3^ (<0.5×10^9^/L) in participants discontinuing fumarate-based therapies
  - Serum IgG <500 mg/dL (according to central laboratory range)
  - Any other clinically significant laboratory assessment as determined by the investigator (eg, significant anemia, neutropenia, thrombocytopenia, signs of impaired bone marrow function)
- Participants with any of the following neurological/psychiatric disorders prior to first study drug administration:
  - Score “yes” on item 4 or 5 of the Suicidal Ideation section of the Columbia-Suicide Severity Rating Scale if this ideation occurred in the past 6 months, or “yes” on any item of the Suicidal Behavior section, except for the “Non-Suicidal Self-Injurious Behavior” (item also included in the Suicidal Behavior section), if this behavior occurred in the past 2 years
- History of hypersensitivity to the study drug or any of the excipients or to drugs of similar chemical classes

**Assessments and procedures**

*IgG/M*

Total IgG and IgM levels were recorded at screening and at visits 4, 7, 9, 11 and 13 (weeks 4, 24, 48, 72, and 96). Values at each time point were analyzed as means with standard error (SE). The lower limits of normal (LLN) were defined as 5.65 g/L for IgG and 0.4 g/L for IgM.

*Exploratory endpoints*

EDSS

EDSS is a widely used and accepted instrument to evaluate disability status at a given time and is also used longitudinally to assess accumulation of disability in clinical studies. The EDSS is an ordinal scale used for assessing neurological impairment in MS based on a neurological examination and consists of scores in each of 7 functional systems (FS: visual, brain stem, pyramidal, cerebellar, sensory, bowel and bladder, cerebral) and an ambulation score that are then combined to determine the EDSS (ranging from 0 [normal] to 10 [death due to MS]). In this study, EDSS was determined based on neurological examination by the investigator at planned visits (ie, at screening, at baseline, and at visits 7, 9, 11, and 13 [Weeks 24, 48, 72, and 96]) and in case of suspected relapse. Each visit window was analyzed as a mean change from baseline, and only participants with a value at both baseline and that visit window were included in the analysis. Clinically relevant changes were considered to be an increase of ≥1.0 point from baseline for participants with baseline EDSS ≤5.5 or ≥0.5 point for those with baseline EDSS >5.5 [34].

MRI

All participants underwent MRI scanning of the brain at screening (baseline) and at visits 7, 9, and 13 (Weeks 24, 48, and 96). MRI scheduling could be adjusted due to the following restrictions: (1) In case of relapse, if an MRI had been scheduled within 14 days of the initiation of steroid treatment, MRI (with Gd+ enhancement) was to be performed before steroid treatment was initiated; (2) No MRI (with Gd+ enhancement) was to be performed while a participant was receiving steroids for relapse or within the following 14 days upon termination of steroid treatment. MRI scan sequences included conventional MRI measures of T1 hypointense images with and without contrast medium (eg, gadolinium-diethylenetriaminepentaacetic acid) and T2 weighted images. Each visit window was analyzed as a mean change from baseline (postbaseline minus baseline), and only participants with a value at both baseline and that visit window were included in the analysis.

Biomarkers

Biomarker sampling glial fibrillary acidic protein (GFAP) took place at screening, visit 4 (Week 4) and subsequently at all other visits from Week 12; additional samples were collected in case of an unscheduled visit due to a suspected MS relapse. If the scheduled visit coincided with the day that study drug administration was scheduled, the participant was instructed not to administer the injection before coming to the site so that the assessments could be completed prior to study drug administration. Postbaseline geometric means of NfL concentrations with 95% confidence intervals were calculated at each visit window. The mixed model for repeated measures on log-transformed change from baseline NfL was fitted, with last prior DMT and time points as factors and baseline age and the log-transformed NfL baseline concentration as continuous covariates. Geometric means at baseline were calculated by exponentiating the arithmetic means of the natural log-transformed raw NfL values (**Online Resource Figure 7**).

***Additional exploratory endpoints***

The T25FW test, 9-HPT, SDMT, and LCVA scores were assessed at baseline and at scheduled follow-up visits. T25FW and 9-HPT were measured at baseline and at Weeks 24, 48, 72, and 96. SDMT and LCVA were measured at baseline, Year 1 (Week 48), and Year 2 (Week 96). Each visit window was analyzed as a mean change from baseline, with T25FW and 9HPT analyzed as percent mean change ([postbaseline minus baseline) divided by baseline] × 100), and only participants with a value at both baseline and that visit window were included in the analysis. Exploratory endpoints not included in this manuscript were digital assessments (Floodlight [Genentech, Inc., South San Francisco, CA] and actigraphy) and GFAP.

Timed 25-foot walk (T25FW) test

The T25FW test is a reliable measure of ambulation that assesses walking speed in participants with MS. It records the time (in seconds) required to walk 25 feet (7.62 meters) as quickly but safely as possible [35]. A clinically meaningful change is defined as a 20% increase (worsening) or decrease (improvement) in measured time from baseline [36].

9-hole peg test (9HPT)

The 9HPT is a neurological evaluation that measures upper extremity function in participants with MS. It assesses the time (in seconds) required to insert and remove nine pegs using the left and right hands (2 trials per hand). A clinically meaningful change is defined as a 20% increase (worsening) or decrease (improvement) in measured time from baseline [36].

Symbol digit modalities test (SDMT)

SDMT is a specific, sensitive, and widely used test for early detection of changes in cognitive processing speed in participants with MS. A clinically significant change on a group level has been defined as a 4-point difference in SDMT raw score or a difference of 10% compared with the previous assessment [37].

Low-contrast visual acuity (LCVA) scores

LCVA assesses visual function by measuring the ability to discern letters at reduced contrast levels (2.5%). A 2-letter increase (improvement) or decrease (worsening) from baseline is considered clinically meaningful [38].

*PROs*

TSQM, MSIS-29, and FSMC scores were assessed at baseline and scheduled follow-up visits and were collected electronically via a handheld device. In the event the questionnaires were unavailable for the participants during a visit (eg, due to technical issue with the handheld), a web backup option was available. Paper versions for PROs were unavailable for this study. For all PROs, assessments were mapped to visit number rather than exact date due to timestamp discrepancies from electronic device issues. Only scheduled visits were included; unscheduled visits were excluded from PRO analyses.

Multiple Sclerosis Impact Scale (MSIS-29)

MSIS-29 is a 29-item self-administered questionnaire used to assess health-related quality of life, including both the physical and psychological impact of MS [39]. Responses (version 2) reflected participants’ views on the impact of MS on their daily lives over the past 2 weeks and were captured on a 4-point scale ranging from “not at all” (1) to “extremely” (4). Physical and psychological domain scores were calculated according to scoring guidelines and transformed to a 0 to 100 scale using the standard formula: 100 × (observed score minus lowest possible score) divided by (highest possible score minus lowest possible score), where higher scores indicate greater disease impact. If more than half of the items in a domain were missing, the domain score was set to missing; otherwise, the mean of the completed items multiplied by the number of items in the scale was used to compute the score. An 8-point change for the physical domain and 6-point change for the psychological domain were considered clinically meaningful thresholds [39].

Treatment Satisfaction Questionnaire for Medication (TSQM)

The TSQM is a validated tool used to psychometrically evaluate patient satisfaction with the study drug (ofatumumab) [40]. The TSQM (version 1.4) comprised 14 items across four domains focusing on effectiveness (3 items), side effects (5 items), convenience (3 items), and global satisfaction (3 items) with ofatumumab treatment over the previous 2 to 3 weeks or since the participant’s last use. Item 4 (medication side effects) was excluded from scoring because adverse events were captured through the AE reporting process. With the exception of item 4, all items use 5- or 7-point response scales scored from least to most satisfied. The 7-point scales include a nonneutral midpoint that provides more positive than negative response options to increase precision at the higher end of the satisfaction range. Item scores were summarized to give four domain scores, which were then transformed to a scale of 0 to 100, with higher scores indicating greater treatment satisfaction and perceived improvement.

Fatigue Scale for Motor and Cognitive Functions (FSMC)

FSMC is a 20-item self-administered questionnaire used to assess fatigue in participants with MS, capturing both motor and cognitive components [41]. FSMC sensitivity and specificity scores were calculated according to validated procedures, with higher scores indicating greater fatigue severity [41].

**
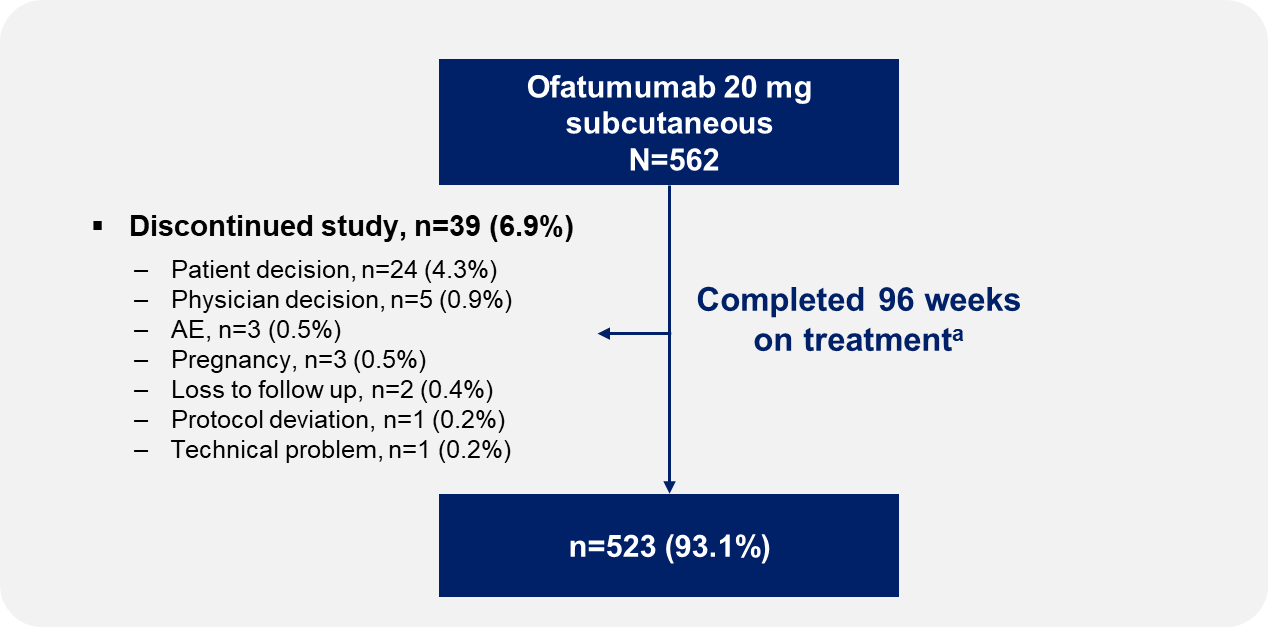
Online Resource Figures**

**Online Resource Fig. 1 Patient disposition**

^a^ Participants were considered to have completed the study if they completed their Week 96 visit. For all percentages, the denominator is the total number of participants (N) for the treatment group.

AE, adverse event.


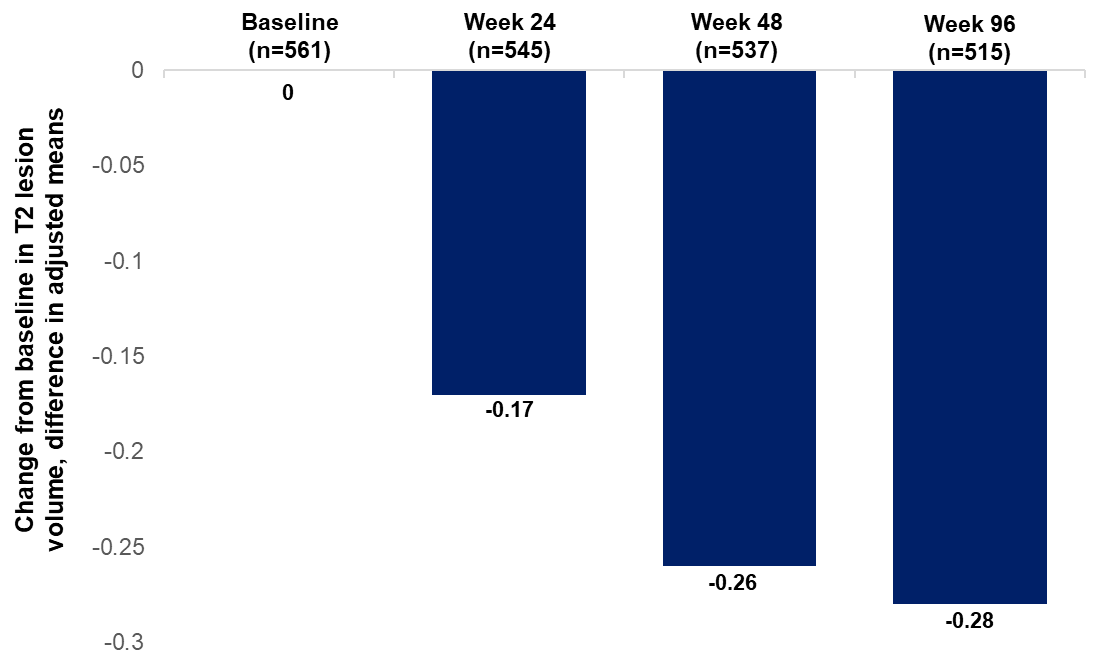
**Online Resource Fig. 2 T2 lesion load in the overall population**

Change from baseline in T2 lesion volume over 96 weeks across all participants.


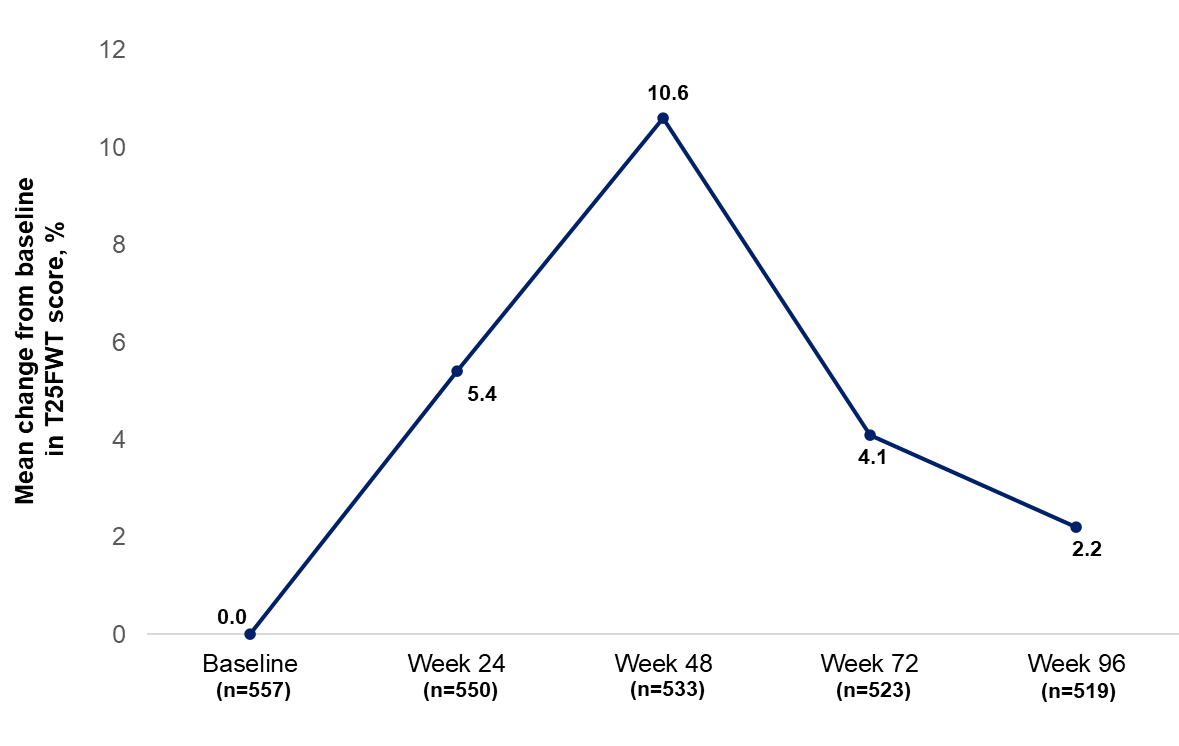
**Online Resource Fig. 3 Percentage change from baseline in TF25W score in the overall population**

At each visit, only participants with a value at both baseline and that visit were included. Clinically relevant T25FW change is defined as a ≥20% increase or decrease from baseline [36].

T25FWT, timed 25-foot walk test.


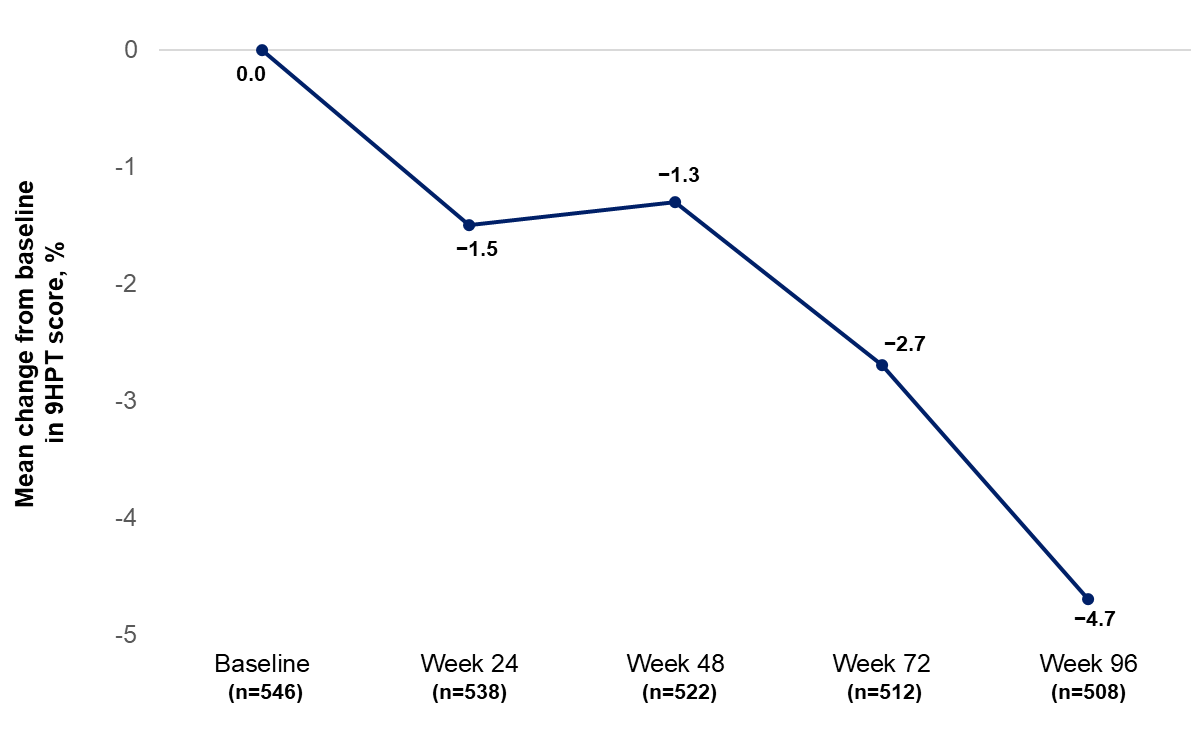
**Online Resource Fig. 4 Percentage change from baseline in 9HPT score in the overall population**

At each visit, only participants with a value at both baseline and that visit were included. Clinically relevant 9HPT change is defined as a ≥20% increase or decrease from baseline [36].

9HPT, 9-hole peg test.

**
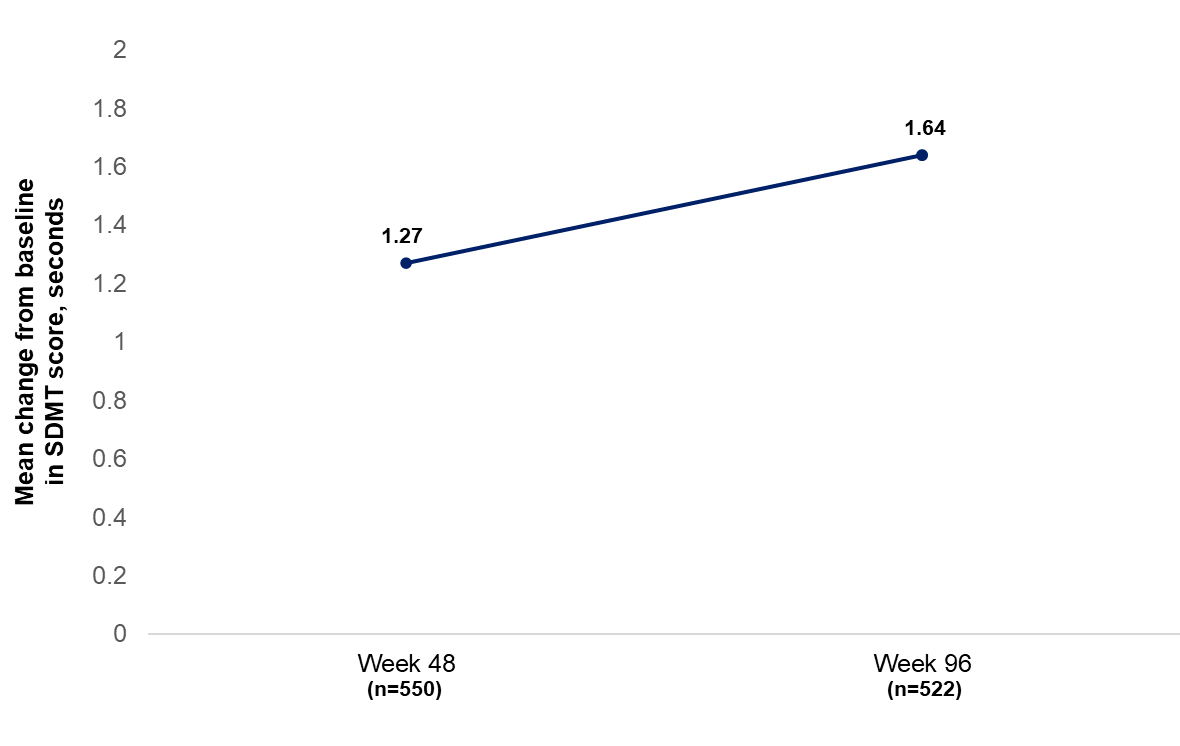
Online Resource Fig. 5 Change from baseline in SDMT score in the overall population**
At each visit, only participants with a value at both baseline and that visit were included. Clinically relevant SDMT change is defined as a ≥10% difference compared with previous assessment [37].

SDMT, symbol digit modalities test.

**
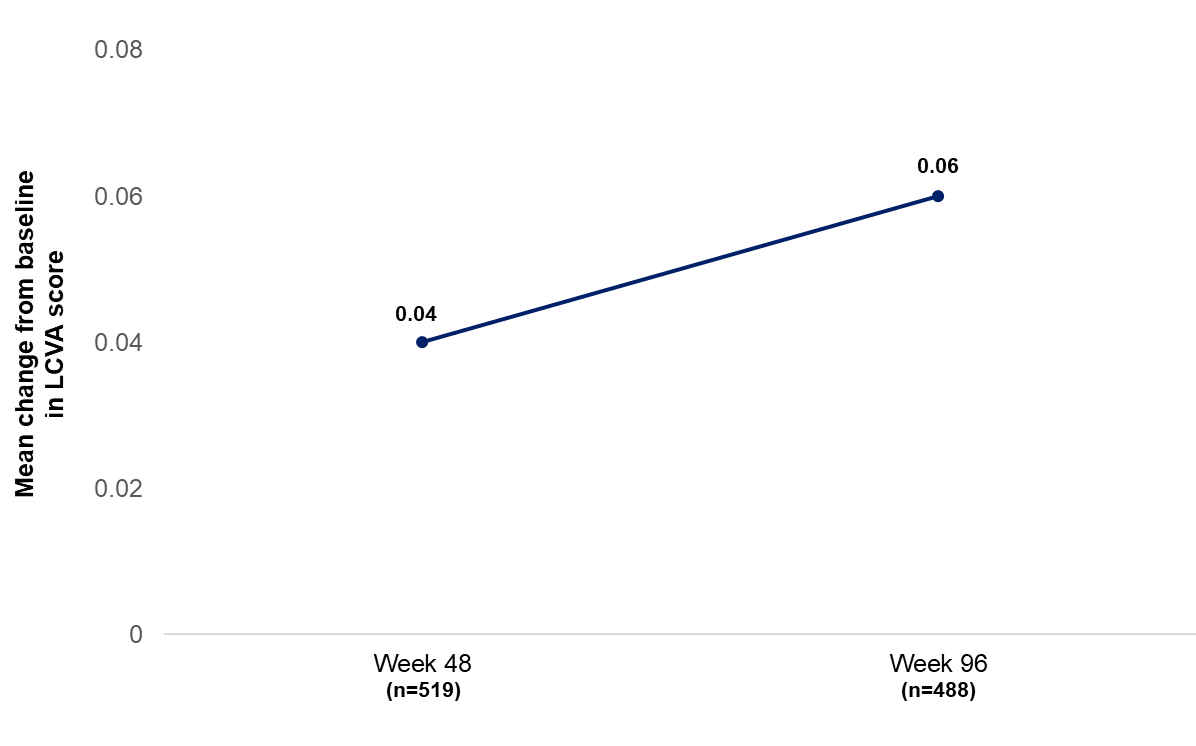
Online Resource Fig. 6 Change from baseline in LCVA in the overall population**
At each visit, only participants with a value at both baseline and that visit were included; only measurements using 2.5% contrast chart were used. Logarithm of the minimum angle of resolution (logMAR) was used to assess LCVA and it was calculated as log10(Snellen VA denominator/Snellen VA numerator). Clinically relevant LCVA change is defined as a change in LCVA scores reflecting a ≥2-letter increase or decrease from baseline [38].

LCVA, low-contrast visual acuity test.

**
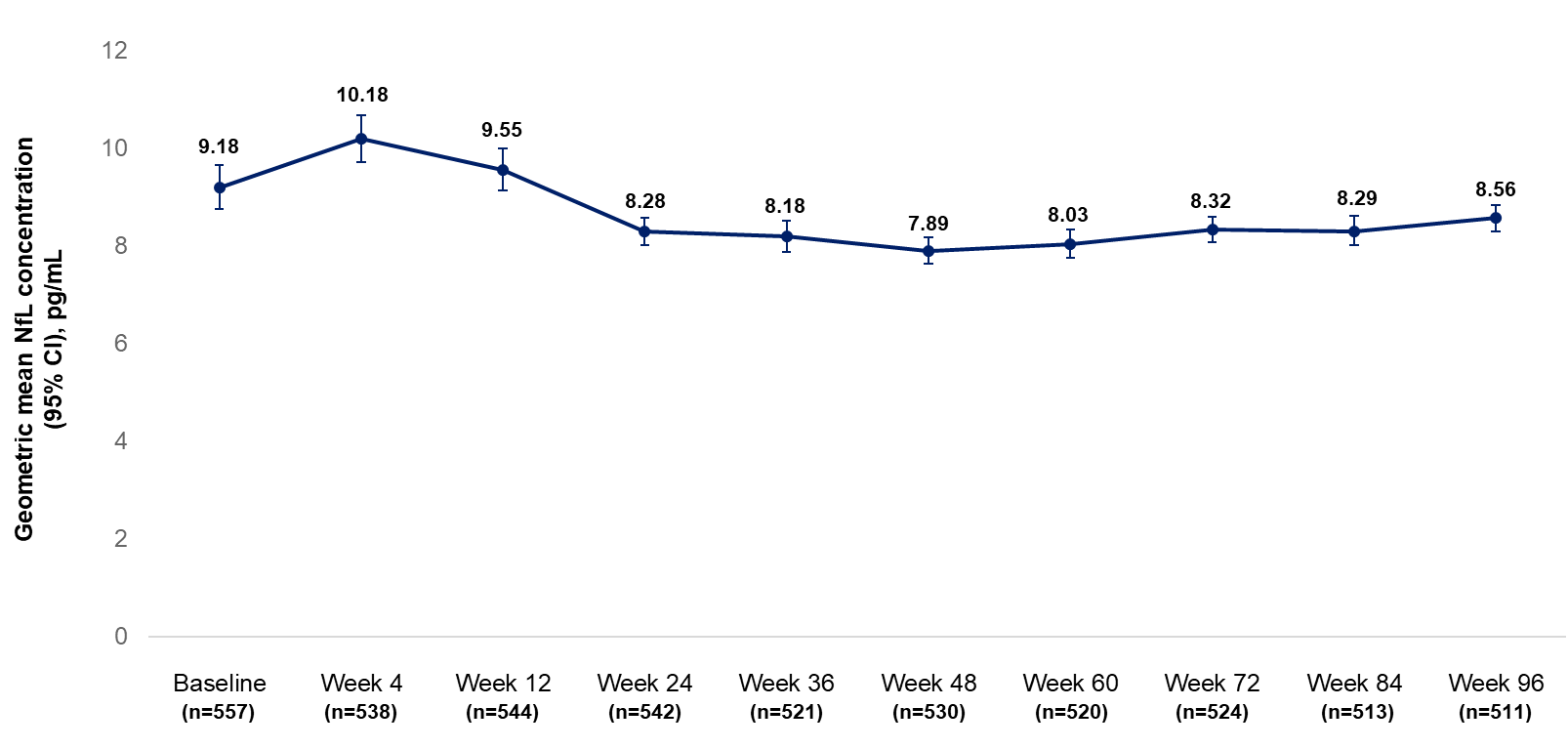
Online Resource Fig. 7 NfL concentration in the overall population**

The adjusted geometric mean and its 95% CI were estimated from fitting a repeated measures model using log-transformed NfL (excluding baseline) as the outcome, with prior DMT and visit as factors, the participants age at baseline and log-transformed NfL baseline concentration as covariates. The NfL geometric mean at baseline was calculated by exponentiating the arithmetic means of the natural log-transformed raw NfL values.
DMT, disease-modifying therapy; NfL, monofilament light chain.

**
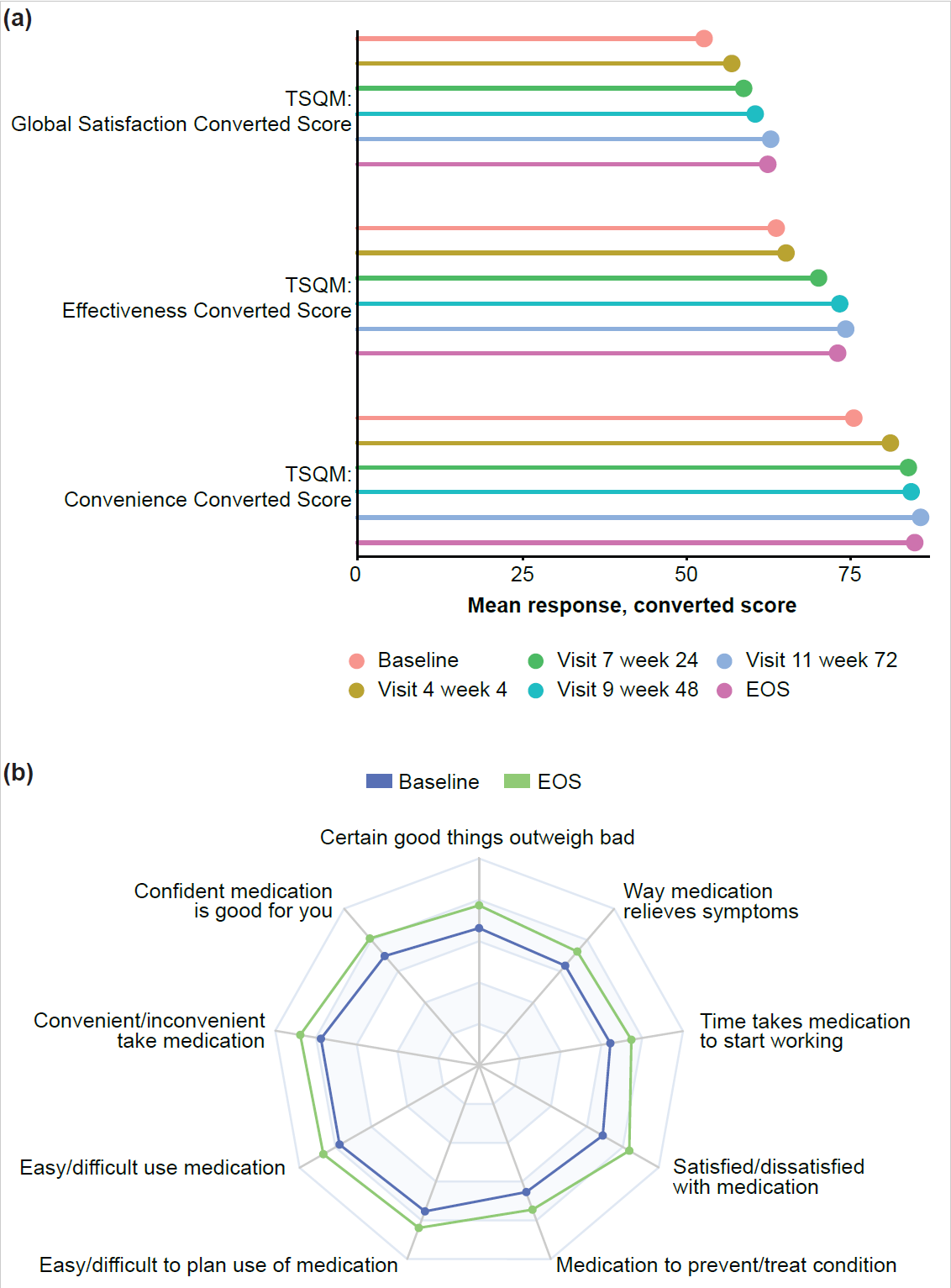
**

**Online Resource Fig. 8 Treatment satisfaction questionnaire for medication scores in the overall population**

(**a**) Lollipop plot including effectiveness, convenience, and global satisfaction domains of the TSQM 1.4. Side effect domains are not included. Domain scores were converted to a 0 to 100 scale. (**b**) Radar plot, including effectiveness, convenience, and global satisfaction domains of TSQM 1.4. Side effect domains are not included. Scores of individual items were standardized, and only baseline and EOS visits were included.

EOS, end of study; med, medication; TSQM, treatment satisfaction questionnaire for medication.

**
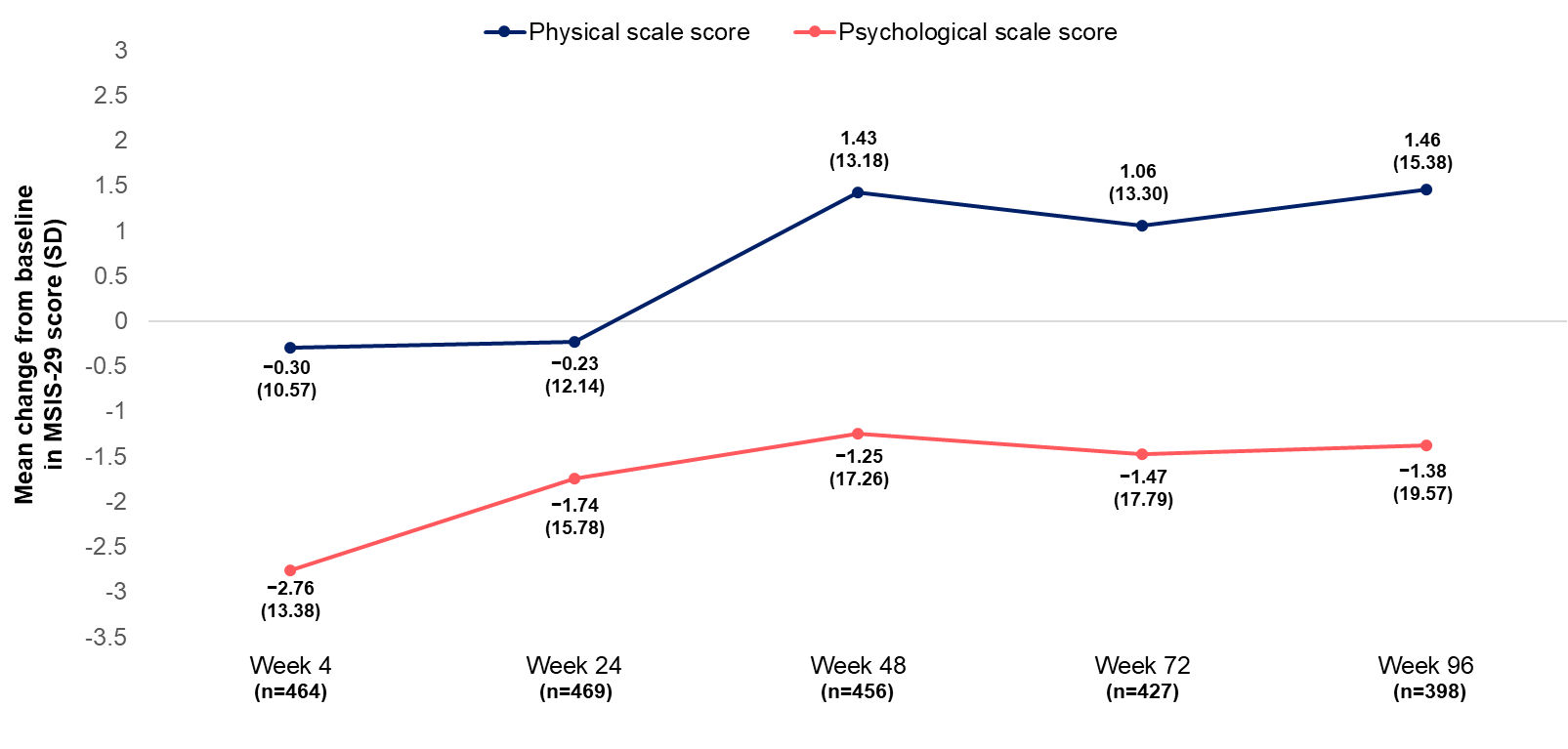
Online Resource Fig. 9 Change from baseline in MSIS-29 in the overall population**

At each visit, only participants with a value at both baseline and that visit were included. Clinically relevant MSIS-29 changes are defined as an ≥8-point difference for the physical domain and ≥6-point difference for the psychological domain from baseline [39].

MSIS, Multiple Sclerosis Impact Scale; SD, standard deviation.


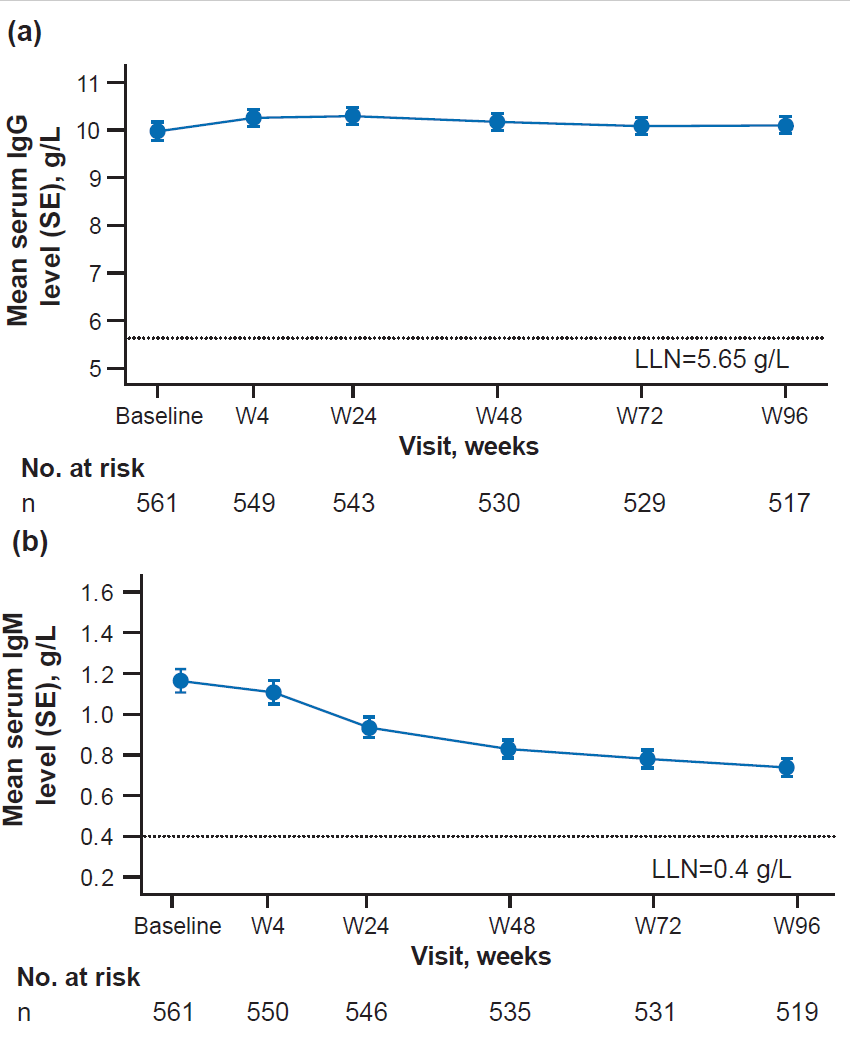
**Online Resource Fig. 10 Serum IgG and IgM levels in the overall population**

(**a**) Mean serum IgG levels. (**b**) Mean serum IgM levels from baseline through Week 96 of ofatumumab treatment.

Ig, immunoglobulin, LLN, lower limit of normal; SE, standard error of the mean.

**
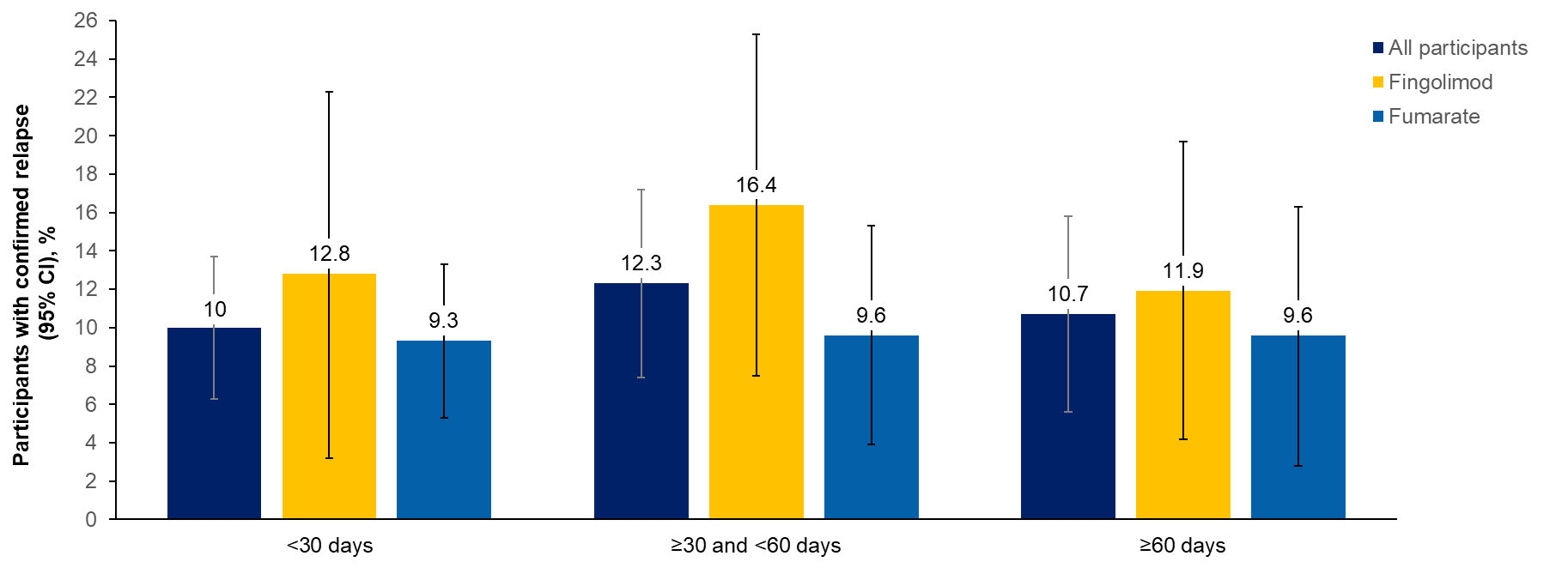
Online Resource Fig. 11 Confirmed relapses by previous DMT and washout period**

Confirmed relapses are those accompanied by a clinically relevant change in the EDSS. Percentages were calculated as the number of participants who had a confirmed relapse in the corresponding washout category and time interval divided by total number of participants in corresponding washout category (n/N).

DMT, disease-modifying therapy; EDSS, Expanded Disability Status Scale.

**
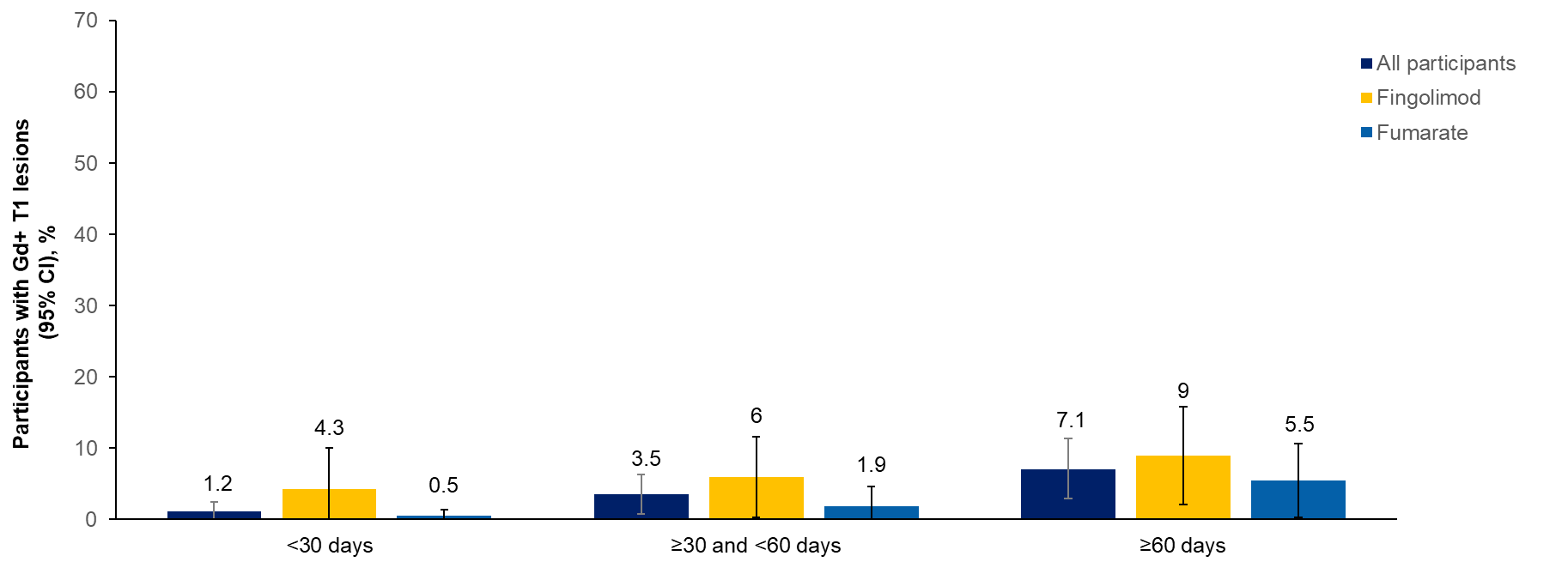
Online Resource Fig. 12 Gd+ T1 lesions by previous DMT and washout period**

Percentages were calculated as the number of participants who had Gd+ T1 lesions in corresponding washout category and time interval divided by total number of participants in corresponding washout category.

DMT, disease-modifying therapy; Gd+; gadolinium enhancing.

**
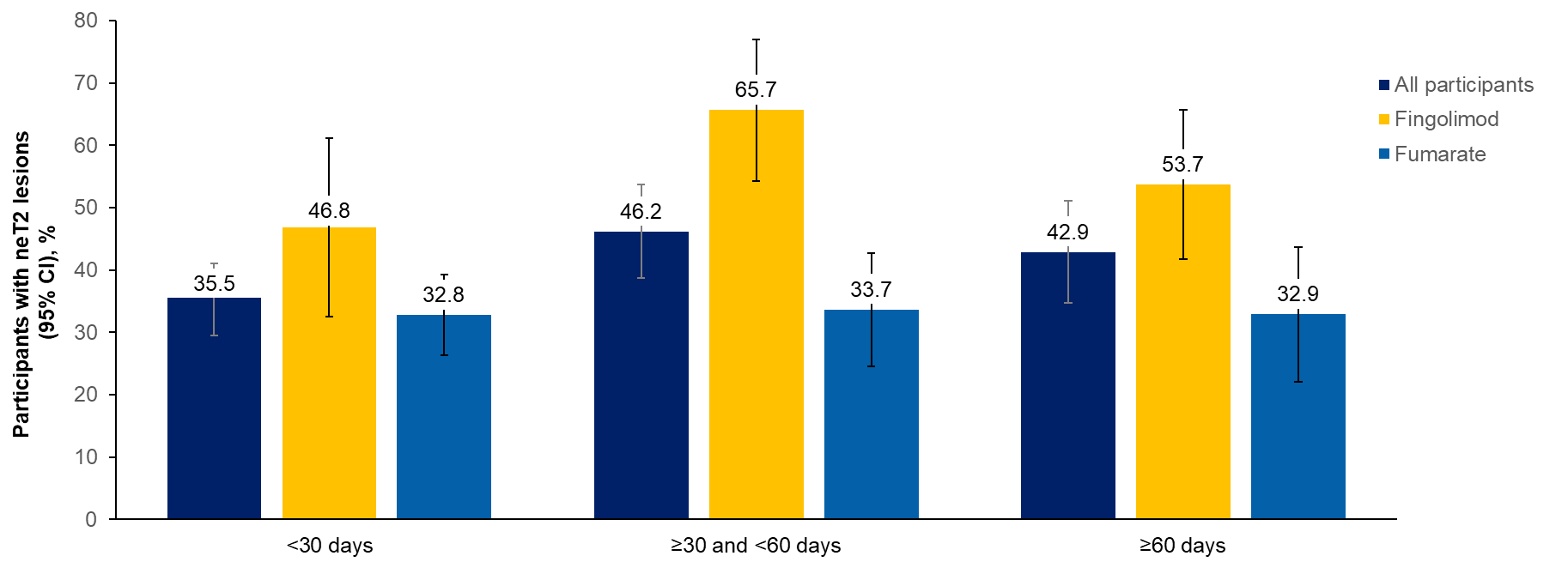
Online Resource Fig. 13 New or enlarging T2 lesions by previous DMT and washout period**

Percentages were calculated as the number of participants who had new or enlarging T2 lesions in corresponding washout category and time interval divided by total number of participants in corresponding washout category.

DMT, disease-modifying therapy; neT2, new or enlarging T2.

**
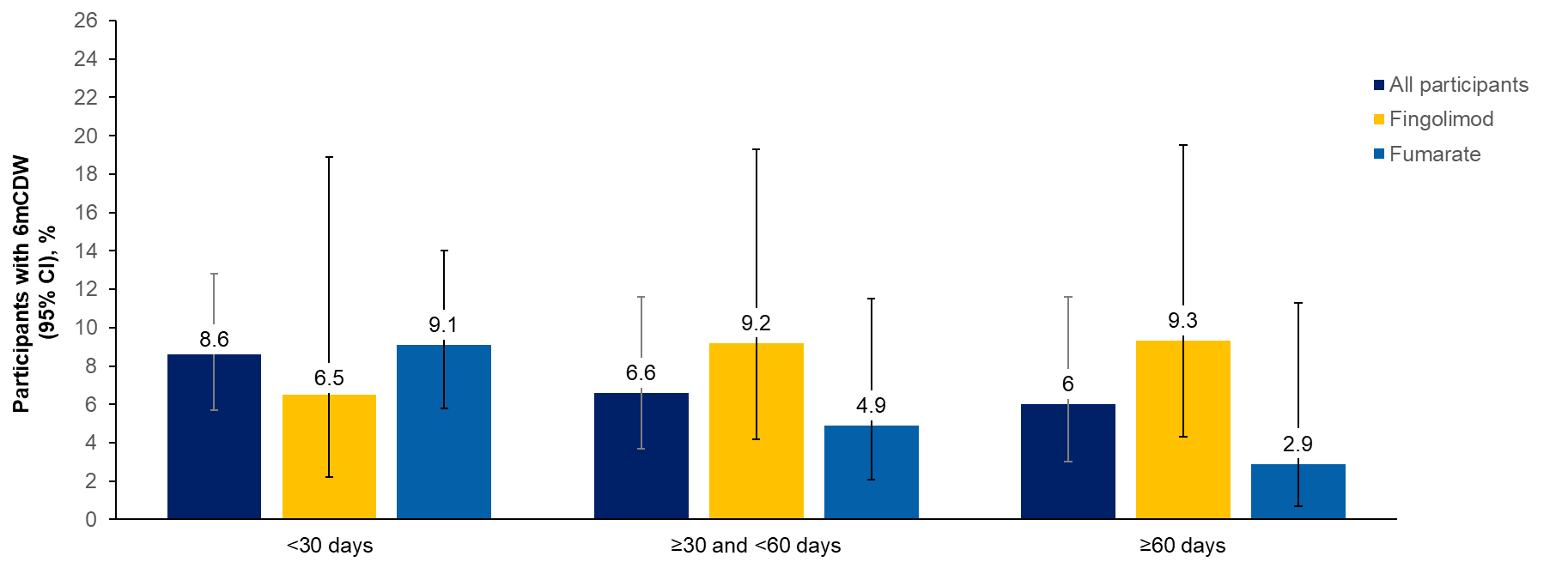
Online Resource Fig. 14 6mCDW by previous DMT and washout period**

6mCDW, 6-month confirmed disability worsening; DMT, disease-modifying therapy.

**
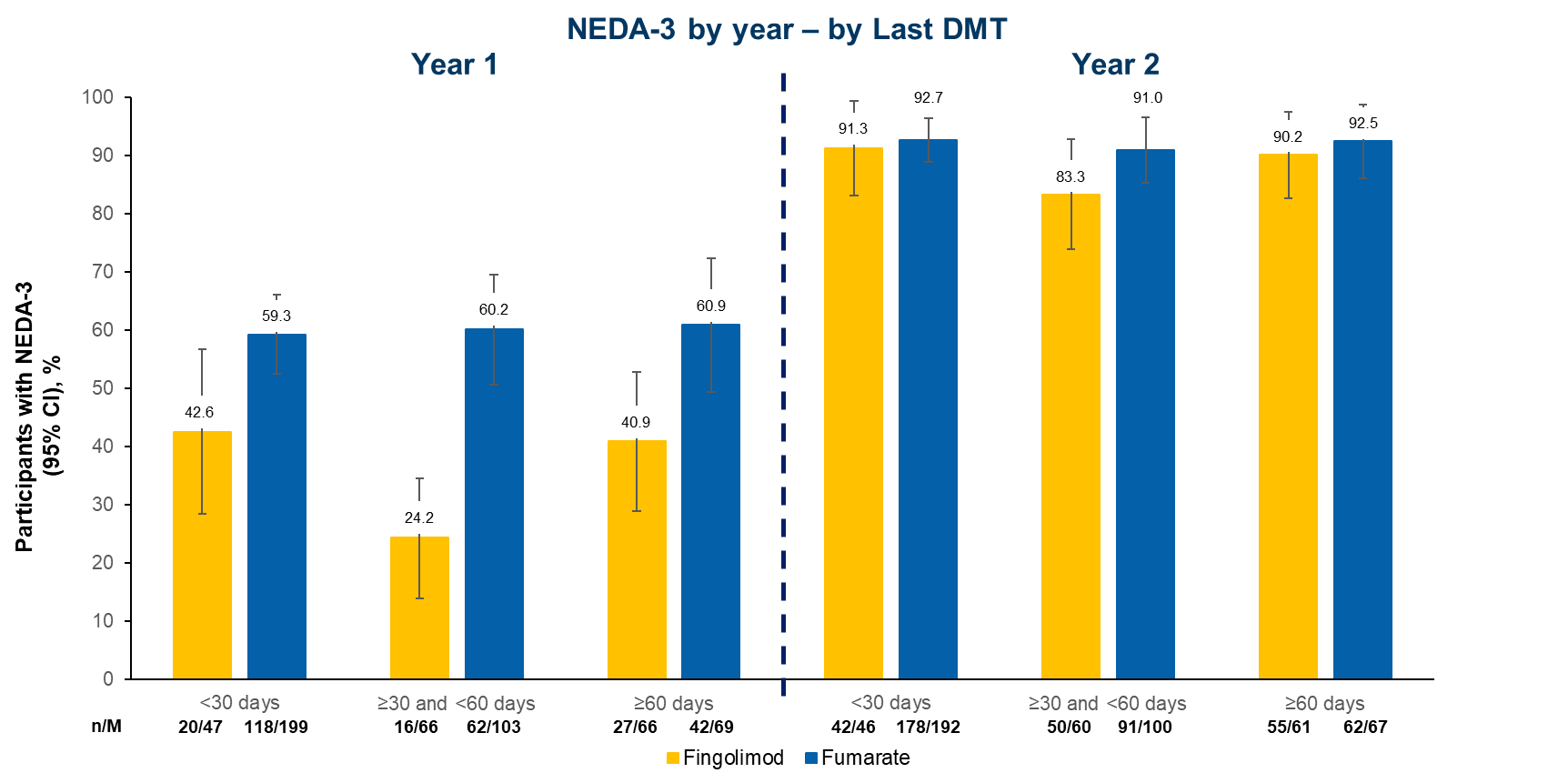
Online Resource Fig. 15 NEDA-3 at Year 1 and 2 by previous DMT and washout p**

NEDA-3 is defined as no 6-month confirmed disability worsening, no confirmed MS relapse, no new or enlarging T2 lesions compared with baseline, and no T1 Gd-enhancing lesions.

DMT, disease-modifying therapy; Gd, gadolinium; M, total number of participants in the treatment group with response variable defined; MS, multiple sclerosis; n, number of participants who responded; NEDA-3, no evidence of disease activity.

**
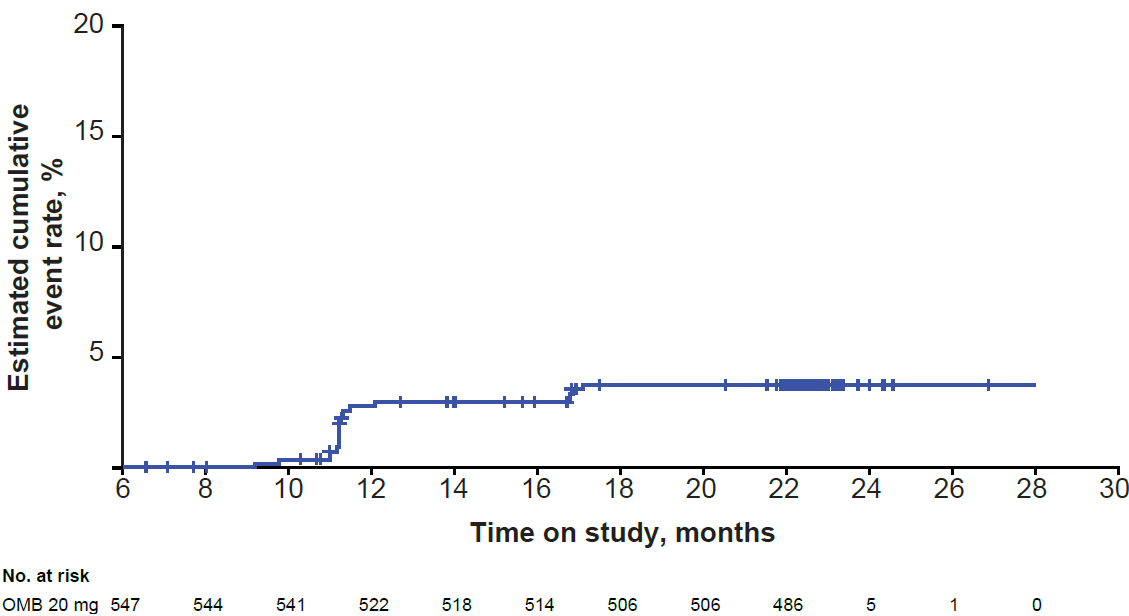
Online Resource Fig. 16 Time to 6mCDW over 96 weeks rebaselined at Month 6 in the overall population**

KM, Kaplan-Meier; OMB, ofatumumab.

**Online Resource Tables**

| **Ethics Committee or  Institutional Review Board** | **City, State/Province, Country** |
| --- | --- |
| 19 Mayis University Medical Faculty | Samsun, Turkey |
| Advarra IRB | Columbia, Maryland, United States |
| AHEPA Thessaloniki University General Hospital | Thessaloniki, Greece |
| Ascension Health | St. Louis, Missouri, United States |
| CEIC - Comissão de Ética para a Investigação Clinica (National Ethics Committee for Clinical Research) | Lisbon, Portugal |
| Centro de Investigación Clínica Gramel, S.C. | Mexico City, Mexico |
| Christiana Care Institutional Review Board | Newark, Delaware, United States |
| Cleveland Clinic IRB | Cleveland, Ohio, United States |
| Cleveland Clinic IRB OS-1 | Cleveland, Ohio, United States |
| Comité de Bioética del Instituto de Investigaciones Clínicas Rosario | Rosario Santa Fe, Argentina |
| Comité de Ética en Investigación Biomédica | Santa Fe, Argentina |
| Commissie voor Medische Ethiek UGent/ UZ Gent | Gent, Belgium |
| EC of Leningradskiy Regional Clinical Oncology Dispansary (LRCOD) | St-Petersburg, Russia |
| Egészségügyi Tudományos Tanács | Budapest, Hungary |
| Ethics Committee for Clinical Research at Pauls Stradins Clinical University Hospital Development Society | Riga, Latvia |
| Ethics Committee for Medicinal Products | Tartu, Estonia |
| Ethics Committee for Multicenter Trials | Sofia, Bulgaria |
| Ethics Committee-Hotel-Dieu de France Hospital | Beirut Achrafieh, Lebanon |
| Ethikkommission der Landesärztekammer Brandenburg | Cottbus, Germany |
| Ethikkommission der Stadt Wien | Vienna, Austria |
| Ethikkommission Nordwest- und | Basel, Switzerland |
| Etická komise | Hradec Králové, Czech Republic |
| Etická komisia FN Nitra | Nitra, Slovakia (Slovak Republic) |
| Etická komisia FN Trnava | Trnava, Slovakia (Slovak Republic) |
| Etická komisia FNsP F.D.Roosevelta | Banská Bystrica, Slovakia (Slovak Republic) |
| Etická komisia LFUK a UNB Nemocnica Staré Mesto | Bratislava, Slovakia (Slovak Republic) |
| Etická komisia UNB Nemocnica akad. L. Dérera | Bratislava, Slovakia (Slovak Republic) |
| Etická komisia UNB Nemocnica Ružinov | Bratislava, Slovakia (Slovak Republic) |
| Etická komisia UNLP Košice | Košice, Slovakia (Slovak Republic) |
| Fundación de Estudios Farmacológicos y de Medicamentos “Prof. Luis M. Zieher” | Buenos Aires, Argentina |
| Hospital Clínico San Carlos | Madrid, Spain |
| Institutional Review Board | Riyadh, Saudi Arabia |
| Institutional Review Board-American University of Beirut Medical Center | Beirut Hamra, Lebanon |
| Institutional Review Board-Lebanese Hospital Geitaoui-University Medical Center (LHG-UMC) | Beirut Achrafieh, Lebanon |
| Institutional Review Board-Rafik Hariri University Hospital | Beirut Jnah, Lebanon |
| Johannes Kepler Universität Linz Ethikkommission der MED Med Campus I Gebäude ADM | Linz Vienna Austria |
| Johns Hopkins Medicine Institutional Review Board (JH IRB) | Baltimore, Maryland, United States |
| Komisja Bioetyczna przy Sląskiej Izbie Lekarskiej | Katowice, Poland |
| Larissa General University Hospital | Larissa, Greece |
| LEC of the City Clinical Hospital 12 na Buyanov | Moscow, Russia |
| Med. Universität Wien und des AKH | Vienna, Austria |
| Mexico Centre for Clinical Research | Mexico City, Mexico |
| Moscow city Independent ethical committee | Moscow, Russia |
| National Medical Ethics Committee (Komisija Republike Slovenije za medicinsko etiko) | Ljubljana, Slovenia |
| OMRF IRB | Oklahoma City, Oklahoma, United States |
| REK Sør-øst | Oslo, Norway |
| Research Ethics Board of Sunnybrook Health Sciences Centre | Toronto, Canada |
| Research Ethics Committee | Jeddah Makkah, Saudi Arabia |
| Sociedad Administradora de Servicios de Salud S.C. | Morelia, Mexico |
| St. Luke's Hospital | Thessaloniki, Greece |
| Territorial Ethics Committee (CET) Area Sud-Overs Veneto | Piazzale Aristide Stefani, Italy |
| The Alfred Hospital Ethics Committee | Melbourne, Australia |
| University of New Mexico Health Sciences Center | Albuquerque, New Mexico, United States |
| West Midlands - Edgbaston Research Ethics Committee | Stratford London, United Kingdom |

**Online Resource Table 1. List of Independent Ethics Committees (IEC) or Institutional Review Boards (IRB) by location**

| **Time point, n/M (%)** | **Fingolimod** | **Fumarates** |
| --- | --- | --- |
| **Baseline** | 112/175 (64.0) | 285/374 (76.2) |
| **Week 24** | 163/171 (95.3) | 352/359 (98.1) |
| **Week 48** | 164/166 (98.8) | 352/355 (99.2) |
| **Week 96** | 159/162 (98.1) | 343/346 (99.1) |
| **Proportion of participants free of Gd-enhancing T1 lesions at all postbaseline scans**^a^ | 142/153 (92.8) | 321/328 (97.9) |

**Online Resource Table 2. Proportion of participants free of Gd-enhancing T1 lesions (per scan) by last prior DMT (FAS)**

^a^ Only participants with results at Weeks 24, 48, and 96 were included (ie, contributed to M).

DMT, disease-modifying therapy; FAS, full analysis set; Gd, gadolinium; M, number of participants with ≥1 result in the category; n, number of participants free of lesions.

|  | | | | **Cumulative rate of events^d^** | | | |
| --- | --- | --- | --- | --- | --- | --- | --- |
| **Treatment** | **Time interval, months^a^** | **Number of participants at risk^b^** | **Number of participants censored^c^** | **Number of events^c^** | **Cumulative number of events** | **Cumulative event rate, %^e^** | **95% CI for cumulative event rate^e^** |
| **OMB**  **20 mg (N=562)** | 0 to <12 | 562 | 31 | 61 | 61 | 11.4 | (9.0-14.4) |
|  | 12 to <24 | 470 | 465 | 3 | 64 | 11.9 | (9.5-15.0) |
|  | 24 to <36 | 2 | 2 | 0 | 64 | NE | (NE-NE) |

**Online Resource Table 3. Event rate for 6mCCD by time interval using Kaplan-Meier estimate in the overall population**

6mCCD is defined as a 4-point worsening on SDMT.

^a^ Based on the following calculation: (date of event or censoring − treatment start date + 1)/30.

^b^ At the beginning of the time interval.

^c^ Within the time interval.

^d^ At the end of the time interval; cumulative since Day 1.

^e^ The cumulative rate of events: 1 − KM estimate of event-free survival. 95% CI for cumulative events rate is based on the KM estimates using the standard error calculated with Greenwood's formula.

6mCCD, 6-month confirmed cognitive decline; KM, Kaplan-Meier; NE, not evaluable; OMB, ofatumumab; SDMT, symbol digit modalities test.

|  | **Cognitive**  **(N=562)** | **Motor**  **(N=562)** | **Total**  **(N=562)** |
| --- | --- | --- | --- |
| **Mean baseline score for all participants assessed at baseline (n=504)** | 23.5 | 25.9 | 49.4 |
| **Mean baseline score for all participants with post-baseline (EOS) scores (n=390)** | 23.6 | 26.9 | 49.5 |
| **Mean postbaseline (EOS) score (n=390)** | 24.9 | 26.8 | 51.7 |
| **Mean change in score from baseline to EOS (n=390)** | 1.3 | 0.9 | 2.2 |

| **Cut-off values for FSMC scores** | | |
| --- | --- | --- |
| **Cognitive score**  Mild cognitive fatigue, ≥22  Moderate cognitive fatigue, ≥28  Severe cognitive fatigue, ≥34 | **Physical score**  Mild physical fatigue, ≥22  Moderate physical fatigue, ≥27  Severe physical fatigue, ≥32 | **Sum score**  Mild fatigue, ≥43  Moderate fatigue, ≥53  Severe fatigue, ≥63 |

**Online Resource Table 4. Change from baseline in FSMC in the overall population**

FSMC is a 20-item scale developed as a measure of cognitive and motor fatigue; higher scores indicate worsening [41].

N reflects full analysis set; n is the number of participants with assessment available at EOS.
EOS, end of study; FSMC, Fatigue Scale for Motor and Cognitive Functions.

| **All grades (preferred term), n (%)** | **Overall**  **population**  **(N=562)** | **By last prior DMT** | |
| --- | --- | --- | --- |
|  |  | **Fingolimod**  **(n=181)** | **Fumarates**  **(n=381)** |
| Systemic injection-related reactions | 300 (53.4) | 92 (50.8) | 208 (54.6) |
| COVID-19 | 208 (37.0) | 62 (34.3) | 146 (38.3) |
| Nasopharyngitis | 96 (17.1) | 25 (13.8) | 71 (18.6) |
| Headache | 92 (16.4) | 30 (16.6) | 62 (16.3) |
| Upper respiratory tract infection | 77 (13.7) | 15 (8.3) | 62 (16.3) |
| Injection site reaction | 62 (11.0) | 20 (11.0) | 42 (11.0) |
| Urinary tract infection | 59 (10.5) | 24 (13.3) | 35 (9.2) |

**Online Resource Table 5. TEAEs that occurred in ≥10% of participants: overall and by prior DMT (safety set)**

DMT, disease-modifying therapy; TEAE, treatment-emergent adverse event.

| **All grades, n (%)** | **Overall**  **population**  **(N=562)** | **By last prior DMT** | |
| --- | --- | --- | --- |
|  |  | **Fingolimod**  **(n=181)** | **Fumarates**  **(n=381)** |
| **Any SAE** | 33 (5.9)^a^ | 13 (7.2) | 20 (5.2) |
| Suicidal ideation | 2 (0.4) | 1 (0.6) | 1 (0.3) |
| Uterine leiomyoma | 2 (0.4) | – | 2 (0.5) |
| Intervertebral disc protrusion | 2 (0.4) | 1 (0.6) | 1 (0.3) |
| Chronic sinusitis | 1 (0.2) | – | 1 (0.3) |
| *Clostridium difficile* infection | 1 (0.2) | 1 (0.6) | – |
| Abscess limb | 1 (0.2) | 1 (0.6) | – |
| Urinary tract infection | 1 (0.2) | – | 1 (0.3) |
| Adenocarcinoma | 1 (0.2) | 1 (0.6) | – |
| Bladder cancer | 1 (0.2) | – | 1 (0.3) |
| Medullary carcinoma of breast | 1 (0.2) | – | 1 (0.3) |

**Online Resource Table 6. Incidence of SAEs, overall and by prior DMT**

^a^ Other SAEs include single incidence of vertigo, cholelithiasis, atrial septal defect, dengue fever, hand fracture, ulna fracture, arthralgia, osteoarthritis, cerebrovascular disorder, myocardial infarction, headache, MS relapse, optic neuritis, sciatica, acute psychosis, depression, ureterolithiasis, endometrial hyperplasia, intermenstrual bleeding, ovarian hematoma, tachycardia, and abnormal MRI.

DMT, disease-modifying therapy; MRI, magnetic resonance imaging; MS, multiple sclerosis; SAE, serious adverse event.

| **TEAE (preferred term), n (%)** | **Total population  (N=562)** |
| --- | --- |
| **Participants with ≥1 TEAE leading to treatment interruption** | 33 (5.9)^a^ |
| COVID-19 | 23 (4.1) |
| Urinary tract infection | 2 (0.4) |
| Diarrhea | 2 (0.4) |
| **Participants with ≥1 TEAE leading to treatment discontinuation** | 5 (0.9) |
| COVID-19 | 1 (0.2) |
| MRI abnormal^b^ | 1 (0.2) |
| Adenocarcinoma | 1 (0.2) |
| Bladder cancer | 1 (0.2) |
| Medullary carcinoma of breast | 1 (0.2) |

**Online Resource Table 7. TEAEs resulting in ofatumumab treatment interruption or discontinuation in the overall population (safety set)**

^a^ Other TEAEs include single incidences of abdominal pain, abdominal pain upper, Bartholin gland abscess, dengue fever, influenza, laryngitis, oral herpes, orchitis, *Pneumonia legionella* infection, suspected COVID-19 infection, tonsillitis, tracheitis, migraine, and suicidal ideation.

^b^ Abnormal MRI consisted of a lesion initially suspicious for progressive multifocal leukoencephalopathy, later confirmed as new MS lesions, that led to study discontinuation based on investigator judgment.

MRI, magnetic resonance imaging; TEAE, treatment-emergent adverse event.

| Adjusted ARR | Overall population | By Last Prior DMT | |
| --- | --- | --- | --- |
|  |  | **Fingolimod** | **Fumarate** |
| Original dataset, (95% CI) | **N=556**  0.06 (0.05-0.08) | **n=178**  0.09 (0.06-0.13) | **n=378**  0.06 (0.04-0.08) |
| Rebaselined at month 6,  (95% CI) | **N=538**  0.04 (0.03-0.06) | **n=174**  0.04 (0.02-0.07) | **n=364**  0.04 (0.03-0.06) |

**Online Resource Table 8. Number of confirmed relapses: rebaselined at month 6, overall, and by prior DMT**

Confirmed relapses are those accompanied by a clinically relevant change in the EDSS.

ARR, annualized relapse rate; DMT, disease-modifying therapy; EDSS, Expanded Disability Status Scale.

| **Adjusted rate of Gd+ T1 lesions per scan** | **Overall population** | **By last prior DMT** | |
| --- | --- | --- | --- |
|  |  | **Fingolimod** | **Fumarate** |
| Original data set  (95% CI) |  |  |  |
| Week 48 | **N=521**  0.02 (0.01-0.07) | **N=166**  0.03 (0.01-0.12) | **N=355**  0.03 (0.01-0.12) |
| Week 96 | **N=508**  0.02 (0.01-0.04) | **N=162**  0.02 (0.01-0.06) | **N=346**  0.02 (0.01, 0.08) |
| Rebaselined at month 6 (95% CI) |  |  |  |
| Week 48 | **N=513**  0.002 (0.001-0.007) | **N=164**  0.014 (0.002-0.081) | **N=349**  0.003 (0.001-0.015) |
| Week 96 | **N=502**  0.003 (0.000-0.013) | **N=161**  0.019 (0.006-0.059) | **N=341**  0.003 (0.001-0.015) |

**Online Resource Table 9. Gd+ T1 lesions: rebaselined at month 6, overall, and by prior DMT**

DMT, disease-modifying therapy; Gd+, gadolinium enhancing.

| **Adjusted rate of neT2 lesions  per scan** | **Overall population** | **By last prior DMT** | |
| --- | --- | --- | --- |
|  |  | **Fingolimod** | **Fumarate** |
| Original data set (95% CI) |  |  |  |
| Week 48 | **N=537**  0.16 (0.11-0.23) | **n=171**  0.30 (0.16-0.55) | **n=366**  0.08 (0.05-0.15) |
| Week 96 | **N=515**  0.07 (0.05-0.10) | **n=165**  0.12 (0.07-0.21) | **n=350**  0.04 (0.02-0.07) |
| Last scan^a^ | **N=550**  0.07 (0.05-0.10) | **n=178**  0.17 (0.10-0.29) | **n=372**  0.05 (0.03-0.09) |
| Rebaselined at month 6 (95% CI) |  |  |  |
| Week 48 | **N=532**  0.14 (0.09-0.22) | **n=170**  0.36 (0.22-0.58) | **n=362**  0.09 (0.05-0.16) |
| Week 96 | **N=511**  0.06 (0.04-0.10) | **n=164**  0.13 (0.08-0.21) | **n=347**  0.04 (0.02-0.08) |
| Last scan^a^ | **N=544**  0.07 (0.04-0.10) | **n=176**  0.15 (0.09-0.24) | **n=368**  0.05 (0.03-0.09) |

**Online Resource Table 10. New or enlarging T2 lesions: rebaselined at month 6, overall, and by prior DMT**

Adjusted new or enlarging T2 lesion is relative to the previous scan.

^a^ The last scan available for the participant.

DMT, disease-modifying therapy; neT2, new or enlarging T2.

| **NEDA-3 at Year 2** | **Overall population** | **By last prior DMT** | |
| --- | --- | --- | --- |
|  |  | **Fingolimod** | **Fumarate** |
| Original data set,  n/M (95% CI), % | 478/526  90.9 (88.4-93.3) | 147/167  88.0 (83.1-92.9) | 331/359 92.2 (89.4-95.0) |
| Rebaselined at month 6,  n/M (95% CI), % | 480/526 (91.3 [88.8, 93.7]) | 146/167  (87.4 [82.4, 92.5]) | 334/359  (93.0 [90.4, 95.7]) |

**Online Resource Table 11. NEDA-3 at Year 2: rebaselined at month 6, overall, and by prior DMT**

NEDA-3 at Year 2 in the original analysis was calculated between baseline and end of study using the end of Year 1 as the cutoff. In the rebaselined analysis, NEDA-3 at Year 2 was recalculated using month 6 as the new baseline for EDSS.

DMT, disease-modifying therapy; EDSS, Expanded Disability Status Scale; M, total number of participants in the treatment group with response variable defined; n, number of participants who responded; NEDA, no evidence of disease activity.

| **Preferred term** **All grades, n (%)^a^** | **Overall population (N=562)** | | **By last prior DMT** | | | |
| --- | --- | --- | --- | --- | --- | --- |
|  |  |  | **Fingolimod**  **(n=181)** | | **Fumarates**  **(n=381)** | |
|  | **<30 days (n=251)** | **≥30 days (n=311)** | **<30 days (n=47)** | **≥30 days (n=134)** | **<30 days (n=204)** | **≥30 days (n=177)** |
| **Participants with ≥1 AE^a^** | 234 (93.2) | 275 (88.4) | 44 (93.6) | 119 (88.8) | 190 (93.1) | 156 (88.1) |
| Injection-related reaction | 147 (58.6) | 153 (49.2) | 22 (46.8) | 70 (52.2) | 125 (61.3) | 83 (46.9) |
| COVID-19 | 76 (30.3) | 132 (42.4) | 11 (23.4) | 51 (38.1) | 65 (31.9) | 81 (45.8) |
| Upper respiratory tract infection | 58 (23.1) | 19 (6.1) | 7 (14.9) | 8 (6.0) | 51 (25.0) | 11 (6.2) |
| Nasopharyngitis | 50 (19.9) | 46 (14.8) | 5 (10.6) | 20 (14.9) | 45 (22.1) | 26 (14.7) |
| Headache | 42 (16.7) | 50 (16.1) | 6 (12.8) | 24 (17.9) | 36 (17.6) | 26 (14.7) |
| Urinary tract infection | 28 (11.2) | 31 (10.0) | 7 (14.9) | 17 (12.7) | 21 (10.3) | 14 (7.9) |
| Injection site reaction | 26 (10.4) | 36 (11.6) | 3 (6.4) | 17 (12.7) | 23 (11.3) | 19 (10.7) |
| **Participants with AE(s) causing study drug discontinuations** | 1 (0.4) | 4 (1.3) | 0 | 3 (2.2) | 1 (0.5) | 1 (0.6) |
| **Participants with AE(s) causing study drug interruptions** | 8 (3.2) | 25 (8.0) | 0 | 11 (8.2) | 8 (3.9) | 14 (7.9) |
| **Participants with ≥1 SAE** | 13 (5.2) | 20 (6.4) | 2 (4.3) | 11 (8.2) | 11 (5.4) | 9 (5.1) |

**Online Resource Table 12. TEAEs that occurred in ≥10% of participants by duration of washout, overall, and by prior DMT (safety set)**

^a^ Only AEs that occurred in ≥10% of the overall population are included.

AE, adverse event; DMT, disease-modifying therapy; N, total number of study participants; n, number of participants in each arm; SAE, serious adverse event; TEAE, treatment-emergent adverse event.
